# Biomarkers as predictors of recurrence of atrial fibrillation post ablation: an updated and expanded systematic review and meta-analysis

**DOI:** 10.1007/s00392-021-01978-w

**Published:** 2022-01-09

**Authors:** Vennela Boyalla, Leanne Harling, Alice Snell, Ines Kralj-Hans, Ana Barradas-Pires, Shouvik Haldar, Habib R. Khan, John G. F. Cleland, Thanos Athanasiou, Sian E. Harding, Tom Wong

**Affiliations:** 1 Royal Brompton and Harefield Hospitals NHS Trust (Part of Guy’s and St Thomas’ NHS Foundation Trust), Sydney Street, London, UK; 2 Imperial College London, London, UK; 3 Department of Thoracic Surgery, Guy’s Hospital, London, UK; 4 Robertson Institute of Biostatistics and Clinical Trials Unit, University of Glasgow, Glasgow, UK; 5 Department of Surgery and Cancer, Imperial College London, London, UK; 6 National Heart and Lung Institute, Imperial College London, London, UK; 7 Kings College London, London, UK

**Keywords:** Atrial fibrillation, Biomarker, Catheter ablation, Outcomes research, Meta-analysis

## Abstract

**Background:**

A high proportion of patients undergoing catheter ablation (CA) for atrial fibrillation (AF) experience recurrence of arrhythmia. This meta-analysis aims to identify pre-ablation serum biomarker(s) associated with arrhythmia recurrence to improve patient selection before CA.

**Methods:**

A systematic approach following PRISMA reporting guidelines was utilised in libraries (Pubmed/Medline, Embase, Web of Science, Scopus) and supplemented by scanning through bibliographies of articles. Biomarker levels were compared using a random-effects model and presented as odds ratio (OR). Heterogeneity was examined by meta-regression and subgroup analysis.

**Results:**

In total, 73 studies were identified after inclusion and exclusion criteria were applied. Nine out of 22 biomarkers showed association with recurrence of AF after CA. High levels of N-Terminal-pro-B-type-Natriuretic Peptide [OR (95% CI), 3.11 (1.80–5.36)], B-type Natriuretic Peptide [BNP, 2.91 (1.74–4.88)], high-sensitivity C-Reactive Protein [2.04 (1.28–3.23)], Carboxy-terminal telopeptide of collagen type I [1.89 (1.16–3.08)] and Interleukin-6 [1.83 (1.18–2.84)] were strongly associated with identifying patients with AF recurrence. Meta-regression highlighted that AF type had a significant impact on BNP levels (heterogeneity *R*^2^ = 55%). Subgroup analysis showed that high BNP levels were more strongly associated with AF recurrence in paroxysmal AF (PAF) cohorts compared to the addition of non-PAF patients. Egger’s test ruled out the presence of publication bias from small-study effects.

**Conclusion:**

Ranking biomarkers based on the strength of association with outcome provides each biomarker relative capacity to predict AF recurrence. This will provide randomised controlled trials, a guide to choosing a priori tool for identifying patients likely to revert to AF, which are required to substantiate these findings.

**Graphical abstract:**

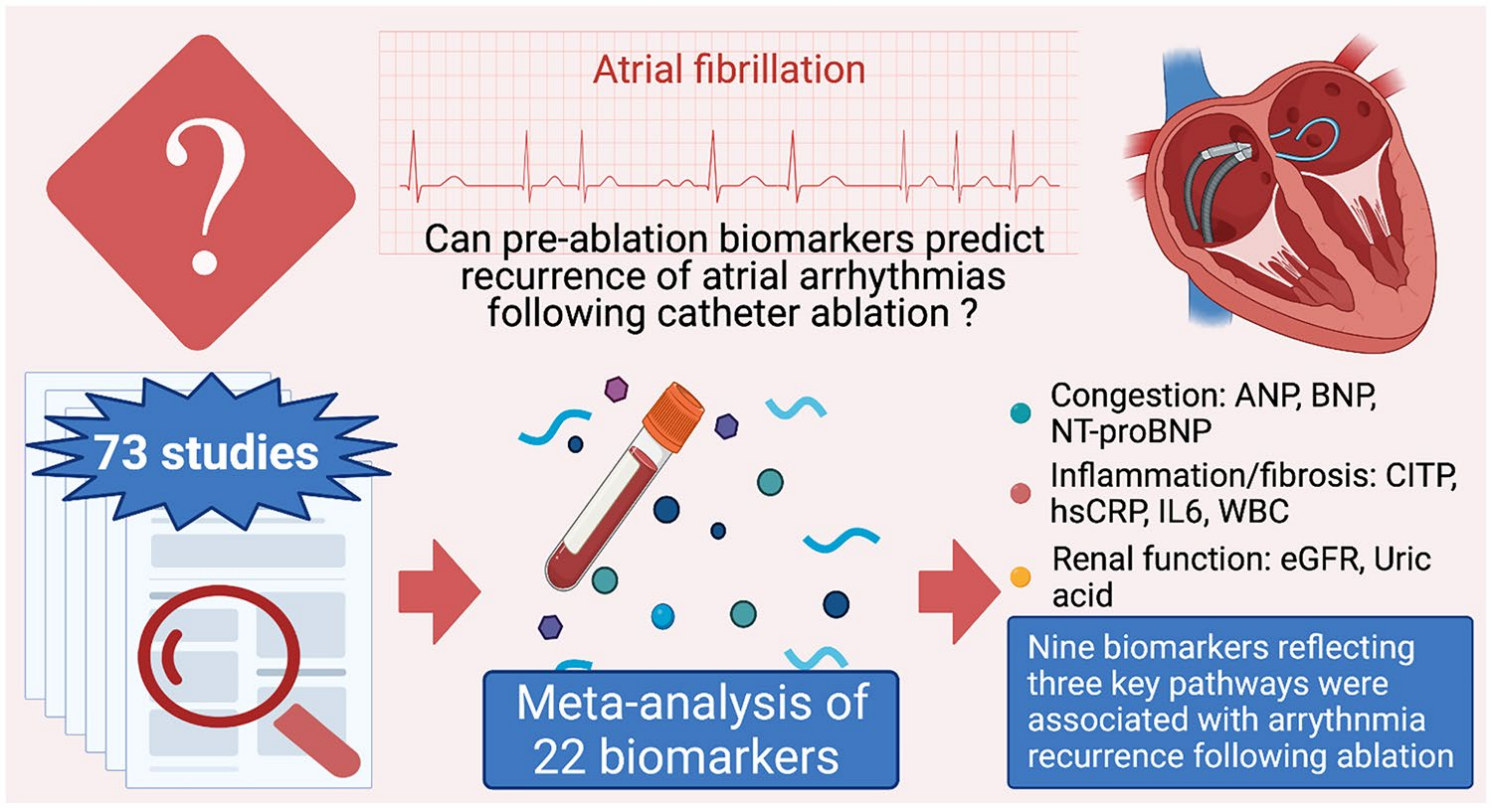

**Supplementary Information:**

The online version contains supplementary material available at 10.1007/s00392-021-01978-w.

## Introduction

Atrial fibrillation (AF) is a major public health concern due to its rising prevalence and associated healthcare impact. An ageing population, multi-morbidity and better survival from other cardiovascular diseases, such as myocardial infarction and heart failure, all contribute to the rise in the incidence of AF [1]. AF generates high health and social care costs due to recurrent health service utilisation for symptom management and associated morbidity (stroke and heart failure) [2]. Catheter ablation (CA) is currently the first-line treatment strategy for rhythm control due to its effectiveness at sustaining sinus rhythm when compared to anti-arrhythmic drugs (AADs [3, 4]. Evidence shows CA improves Quality of Life (QoL), reduces heart failure hospitalisations, stroke and death from cardiovascular causes [5–8]. Yet, long-term ($\ge$ 3 years) freedom from atrial arrhythmias following a single ablation procedure is achieved in only half of the treated patients with paroxysmal and persistent AF [53.1% (95% CI 46.2–60.0%)] [9]. Risk factors, such as advanced age and chronicity of AF, increase the likelihood of recurrence but are not independently predictive of ablation failure. Hence, there is a pressing need for better pre-ablation screening tools, including biomarkers, to identify patients at risk of recurrence following the procedure. Moreover, identifying a blood/serum biomarker can potentially lead to personalised medicine, risk stratification of patients before invasive strategies or novel drug targets.

Natriuretic peptides, C-reactive protein (CRP), interleukin-6 (IL-6), low-density lipoprotein (LDL), tissue inhibitor of metalloproteinase-2 (TIMP) and galectin-3 (Gal-3) were all associated with a greater risk of recurrence following radiofrequency CA in previous meta-analyses [10–13]. In the last 5 years, the number of published papers assessing biomarkers in AF ablation has nearly doubled. Additionally, high-sensitivity assays for CRP (hsCRP), carboxy-terminal telopeptide of collagen type I (CITP), neutrophil/lymphocyte ratio (NLR) have all become of greater interest. Our study is the first to rank baseline blood biomarkers based on their strength of association to AF recurrence following CA, using PRISMA guidelines, resulting in updating and expanding the scope of the previous meta-analysis [12].

## Methods

### Search strategy

This work followed PRISMA guidelines, and the checklist is in the supplementary file (see Table 1 for the checklist). Four search engines were used (PubMed/Medline, Embase, Web of Science, Scopus), and studies published until the end of May 2021 were included. Our broad search strategy is described in Supplementary Table 2. This systematic review involved identifying a test with prediction or prognostic capabilities; therefore, a modified version for PICO(TS) model was used to design the study protocol (see Supplementary Table 3 for model) [14].

### Selection criteria

A *piori screening* criteria to identify appropriate articles are listed in Supplementary Table 3. Systematic reviews and meta-analyses identified in the search were further reviewed to retrieve relevant studies. A minimum of 3 articles for each biomarker were required to be included in this meta-analysis. If multiple publications consisted of overlapping populations, the study with the largest sample size was included in the analysis and the others excluded.

### Data abstraction

One author (V.B.) extracted data independently, and a second author (L.H.) verified the data. The extracted data composed of the following information: (1) title, (2) author name, (3) year of publication, (4) country/region of participant recruitment, (5) Study design, (6) total number of participants with AF, (7) characteristics of participants (mean age, gender proportions, AF type), (8) mean/median follow-up duration, (9) years of recruitment, (10) recurrence rates, (11) type of assessment of AF recurrence using a continuous rhythm recording device, (12) mean, (SD) and median (IQR) values of biomarker(s) for Recurrence (R) and Non-Recurrence (NR) groups. Disagreement was resolved by consensus or adjudication by a third author (H.K.).

### Bias assessment tool

QUality In Prognostic Studies Tool (QUIPS) was used to assess the risk of bias for all the included studies [15], as recommended by Cochrane Prognosis Methods Group. The QUIPS tool consists of six domains. We determined that the overall risk of bias was moderate or high for our analyses, even if only one domain was classed as either moderate or high. The assessments were done independently by two authors (A.S. and I.KH.), with disagreements resolved by consensus with A.BP.

### Statistical analysis

To identify blood biomarkers taken before ablation that can be utilised as independent predictors of atrial arrhythmia recurrence in patients undergoing their first ablation for AF, we compared their levels in recurrence (R) and non-recurrence (NR). Means (SD) or median (range/IQR) of biomarker(s) values in each group were collected. Medians (range/IQR) were converted to means (SD) using mathematical conversions, as per Wan et al. [16]. Standardised difference of the mean was calculated for biomarkers in each study, which was then used to generate odds ratio (OR) for biomarkers (smd2or function in R Studio). This enabled the presentation of pooled OR of studies in forest plots with a 95% confidence interval (CI) using a random-effects model. A random-effects model was chosen due to anticipated heterogeneity in the data. We ranked the biomarkers based on OR and their statistical significance.

The meta-analysis was undertaken using RStudio software (version 1.3.1056). Statistical heterogeneity in the effects size estimates was investigated by *χ*^2^ (with degree of freedom) and *I*^2^-statistic. The quality of data was evaluated by outlier and bias assessments. Meta-regression was conducted on biomarker(s) that have been analysed in 10 or more studies for the effect of year of publication, mean age, sex and AF type. There are 4 AF types: paroxysmal (PAF), persistent AF (PersAF), long-standing persistent AF (LSPAF) and non-PAF (PersAF, LSPAF, or not defined in the paper). This was followed by subgroup analysis to categorise sources of heterogeneity and their impact. Egger’s test and funnel plot were used to assess statistical publication bias. Two authors (V.B. and L.H.) conducted data synthesis, and the discrepancy was resolved after consultation with another author (T.A.). Significance was set at *p* < 0.05.

## Results

### Study selection

**Fig. 1:**
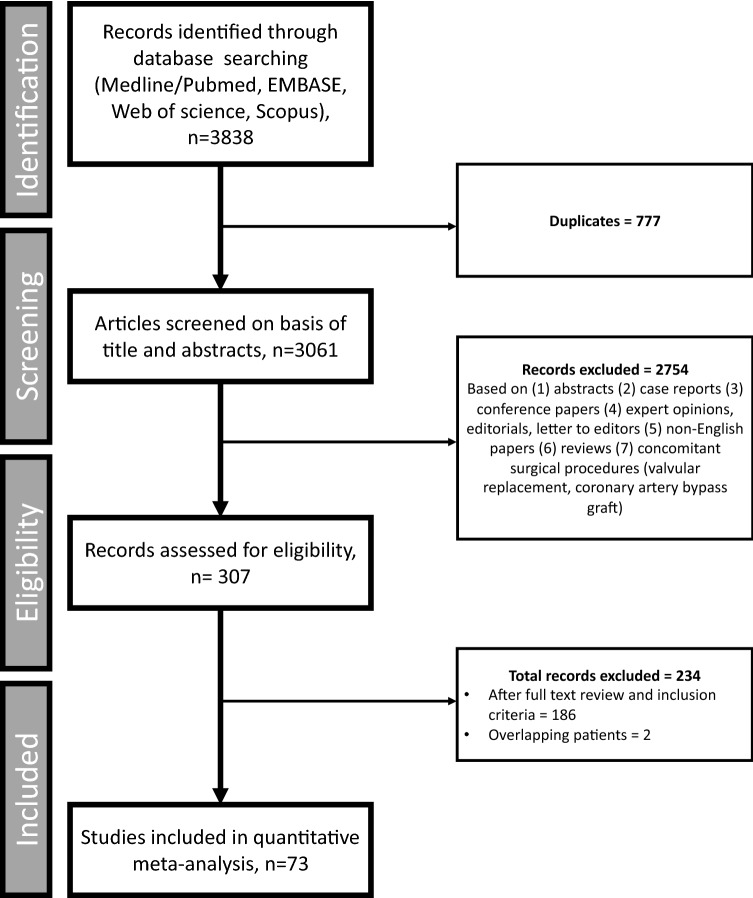
Search strategy and flowchart of study selection in accordance with PRISMA guidelines

The a priori search strategy identified 3061 articles (Fig. 1) after removing duplicates. Post-preliminary screening using abstracts and applying the exclusion criteria, 2754 articles were excluded. The remaining 307 articles were carefully evaluated (full text) utilising the inclusion criteria, leading to further exclusion of 234 studies.

### Study characteristics

A total of 73 studies and 14,148 participants were included, with a mean age of 59 ($\pm$ 10 SD). They were followed up for 3–61 months and AF recurrence rates varied from 12 to 83%. Raw data comprising baseline characteristics and follow-up for individual studies can be found in Supplementary Table 2. Type of ablation (radiofrequency, cryoballoon), strategy (pulmonary vein isolation, linear lesions, mitral isthmus line, complex fractionated atrial electrograms, cavotricuspid isthmus) and proportions of AF type (PAF, persistent AF, LSPAF) from the individual studies are described in Supplementary Table 3. The 73 articles (Supplementary References) included 22 biomarkers:

1. Natriuretic peptides [atrial natriuretic peptide (ANP), brain natriuretic peptide (BNP), N-terminal pro-brain natriuretic peptide (NT-proBNP)],
2. Fibrosis markers [gal-3, CITP, TIMP, transforming growth factor-beta (TGF-β)],
3. Inflammatory pathway markers [tumour necrosis factor-alpha (TNF), CRP, hsCRP, white blood cell (WBC), NLR, IL-6],
4. Lipid profile markers [cholesterol, LDL, high-density lipoprotein (HDL), triglycerides (TG)],
5. Others, such as renal function indicators [creatinine (Cr), eGFR], cardiac injury marker [troponin I (Trop)], uric acid and Haemoglobin A1c (HbA1c).

### Natriuretic peptides and association with AF recurrence

**Fig. 2:**
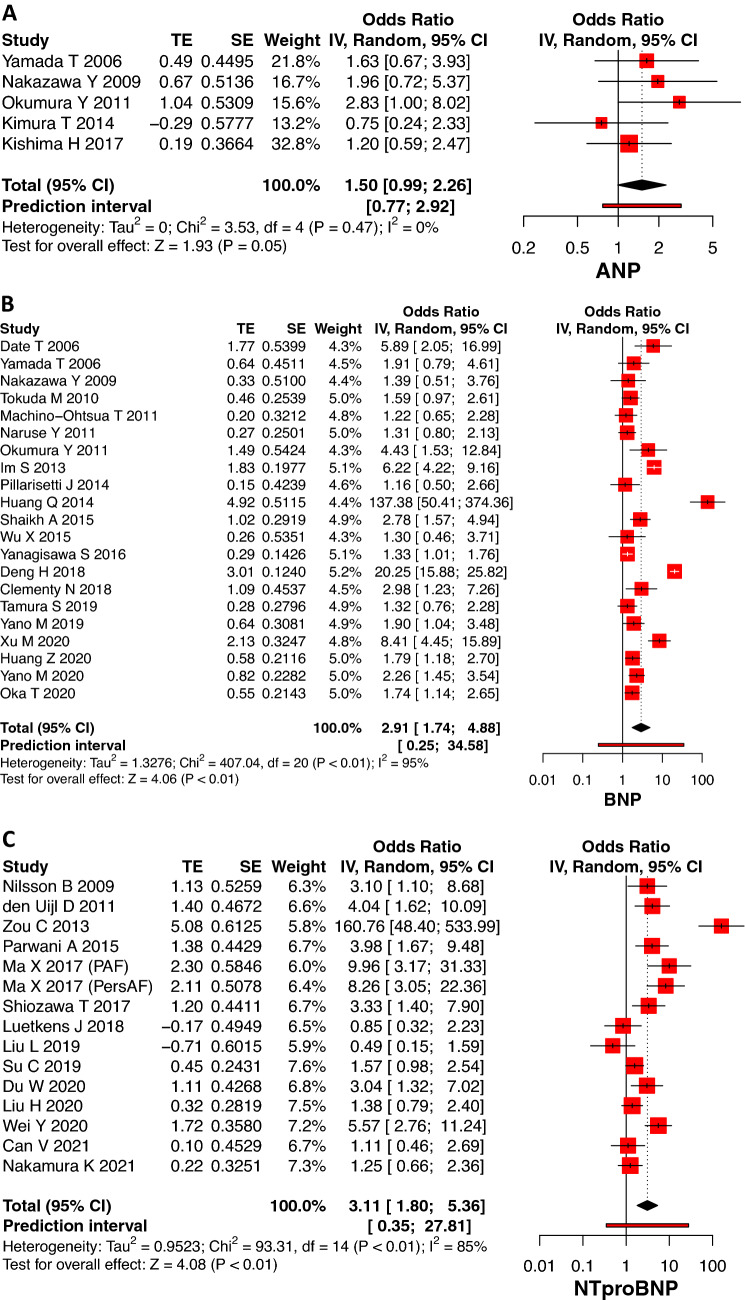
Forrest plot showing association between baseline natriuretic peptides [ANP (**2A**), BNP (**2B**), NT-proBNP (**2C**)] and AF recurrence post ablation. *TE* estimate of effect size, *SE* standard error of effect size, *CI* confidence interval

Based on five studies involving 324 patients, high levels of baseline ANP were significantly associated with AF recurrence post ablation (OR 1.50, 95% CI: 0.99–2.26, *p* = 0.05, Fig. 2). There was no statistical heterogeneity (*I*^2^ = 0) found for these studies, with no outliers detected. There were 21 studies involving 5008 patients in the meta-analysis for BNP, and the pooled result showed that baseline BNP was significantly higher in patients who experienced AF recurrence post CA compared to those that remained in sinus rhythm (OR 2.91, 95% CI: 1.74–4.88, *p* < 0.01, Fig. 2). However, the heterogeneity was significantly high (*I*^2^ = 95%, *p* < 0.01) and remained so even when outliers were removed (*I*^2^ = 78%, *p* < 0.01; Supplementary Fig. 1). Fifteen studies were pooled for assessing baseline NT-proBNP levels in 2165 patients. The recurrence group had significantly higher NT-proBNP than the non-recurrence group following ablation (OR 3.11, 95% CI: 1.80–5.36, *p* < 0.01, Fig. 2). Heterogeneity decreased from 85% to 69% following outlier abstraction. Excluding outliers resulted in diminishing the strength of association of high levels of BNP and NT-proBNP, and AF recurrence (BNP OR 2.14, 95% CI: 1.62–2.83), *p* < 0.01 and NT-proBNP OR 2.63, 95% CI: 1.77–3.91, *p* < 0.01, Supplementary Fig. 1).

### Inflammatory markers and association with AF recurrence

**Fig. 3:**
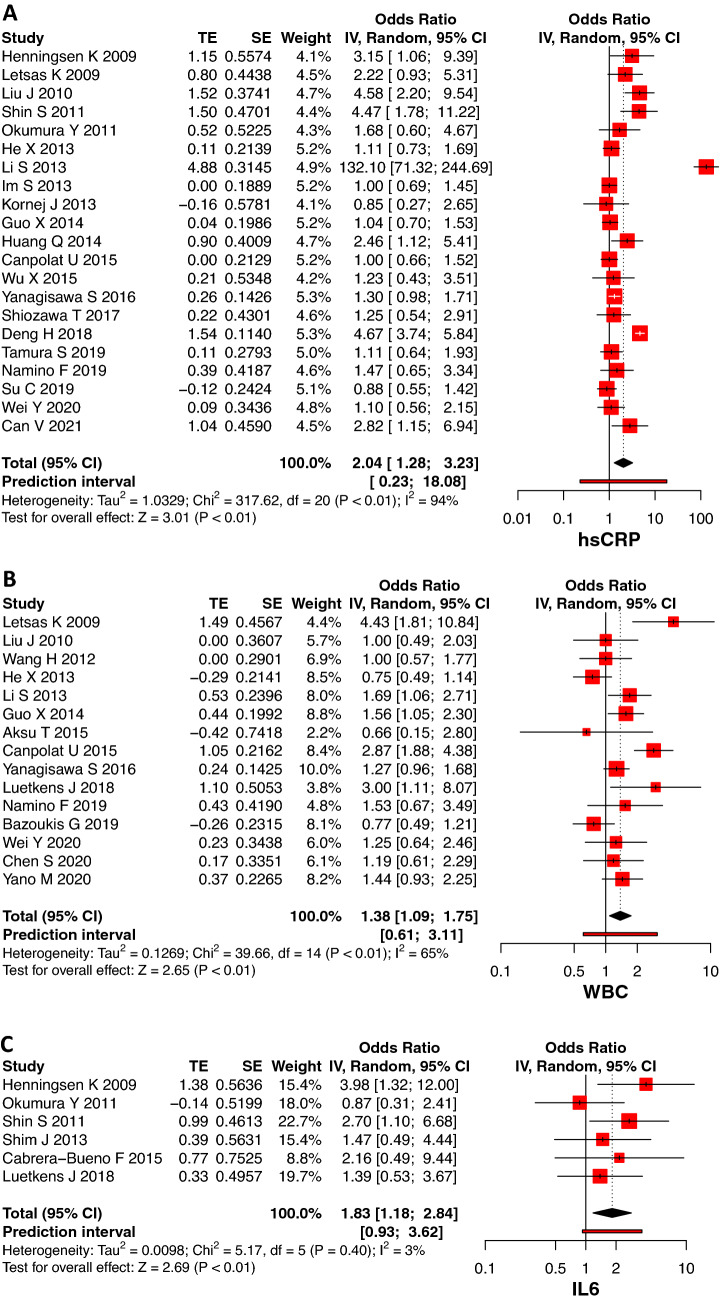
Forrest plot showing an association between baseline inflammatory markers [hsCRP (**3A**), WBC (**3B**), IL-6 (**3C**)] and AF recurrence post catheter ablation. *TE* estimate of effect size, *SE* standard error of effect size, *CI* confidence interval

Based on 21 studies (5049 patients), pooled SMD for baseline hsCRP showed that levels were higher in the recurrence group compared to the non-recurrence group post-ablation (OR 2.04, 95% CI: 1.28–3.23, *p* < 0.01, Fig. 3). The heterogeneity of these studies was high (*I*^2^ = 94%, *p* < 0.01). Subtracting outliers reduced heterogeneity from high to moderate (*I*^2^ = 51%, *p* < 0.01) with a decreased strength of association of hsCRP and AF recurrence (OR 1.40, 95% CI: 1.15–1.72, *p* < 0.01, Supplementary Fig. 1). There were 15 studies retrieved for baseline WBC, and levels were higher in patients with AF recurrence post-CA (OR 1.38, 95% CI: 1.09–1.75, *p* < 0.01, Fig. 3). The heterogeneity of these studies was moderate to high (*I*^2^ = 65%, *p* < 0.01). The magnitude of association reduced from OR 1.38 to OR 1.20 after removing outliers (95% CI: 1–1.44, *p* = 0.05, Supplementary Fig. 1). Six studies showed that baseline IL-6 levels were higher in patients with recurrence of AF following ablation than those that maintained sinus rhythm (OR 1.83, 95% CI: 1.18–2.84, *p* < 0.01, Fig. 3). The studies showed very low heterogeneity (*I*^2^ = 3%, *p* = 0.40). There were no outliers identified in studies reporting IL-6.

### Other biomarkers and association with AF recurrence

**Fig. 4:**
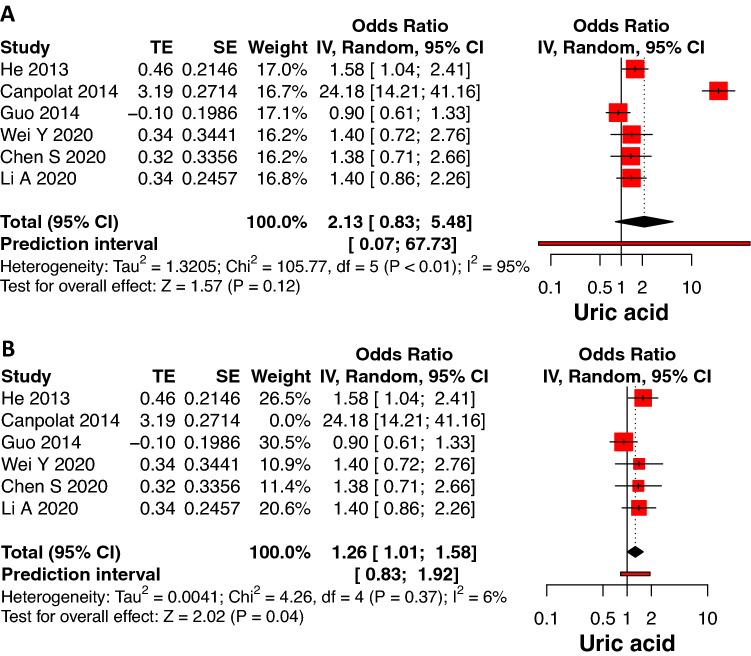
Forrest plot showing the association between baseline uric acid and AF recurrence post catheter ablation with outliers (**4A**) and after removing outliers (**4B**). *TE* estimate of effect size, *SE* standard error of effect size, *CI* confidence interval

**Fig. 5:**
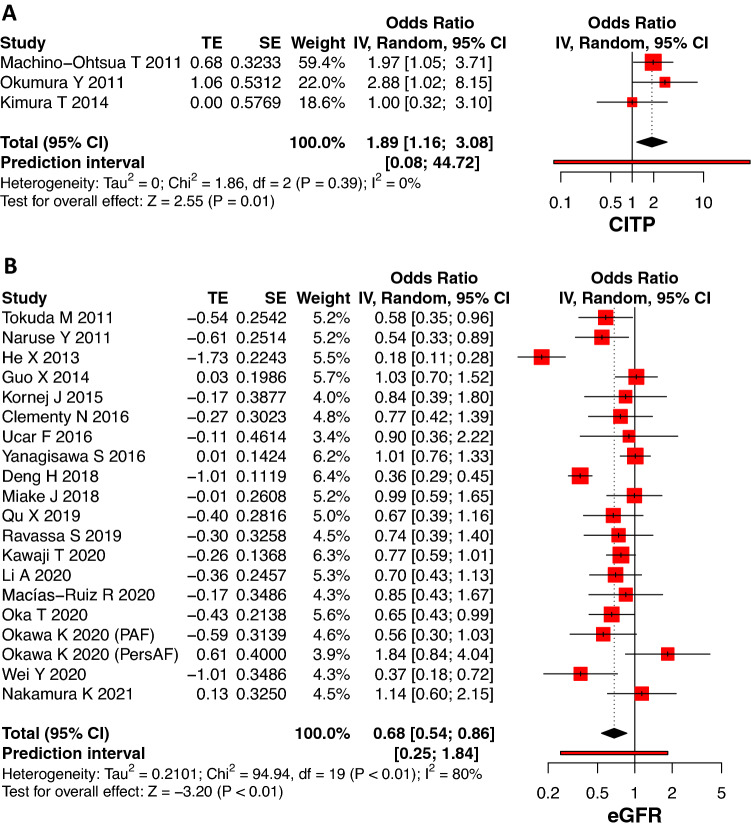
Forrest plot showing the association between baseline CITP (**5A**) and eGFR (**5B**) and AF recurrence post catheter ablation. *SD* standard deviation, *TE* estimate of effect size, *SE* standard error of effect size, *CI* confidence interval

Lipid markers (cholesterol, LDL, HDL and TG), fibrosis/inflammation biomarkers (CRP, NLR, TNF, TGF-β, Gal-3, TIMP), creatinine, troponin I and HbA1c did not show variation in levels between the groups (recurrence vs non-recurrence) following AF ablation (Supplementary Fig. 2, 3, 4, 5). After removing outliers, raised baseline uric acid levels were shown to be associated with AF recurrence following ablation (OR 1.26, 95% CI: 1.01–1.58, *p* = 0.04, Fig. 4). Three studies reported that baseline CITP values were higher in AF recurrence than the non-recurrence group (OR 1.89, 95% CI: 1.16–3.08, *p* = 0.01, Fig. 5). The heterogeneity of these studies was low (*I*^2^ = 0%, *p* = 0.39). Only eGFR was present in low levels in the recurrence group compared to non-recurrence in 19 studies (OR 0.68, 95% CI: 0.54–0.86, *p* < 0.01, Fig. 5). The heterogeneity was high (*I*^2^ = 80%, *p* < 0.01) and decreased after removing outliers (*I*^2^ = 25%, *p* = 0.16; OR 0.78, 95% CI: 0.68–0.90, *p* < 0.01; Supplementary Fig. 1).

### Ranking, meta-regression, sub-group analysis and publication bias

The ranking of biomarkers and their association with AF recurrence was based on pooled ORs. The highest ORs were detected for NT-proBNP (3.11), BNP (2.91), hsCRP (2.04), CITP (1.89) and IL-6 (1.83). Meta-regression analysis was conducted for biomarkers with 10 or more studies (BNP, NT-proBNP, hsCRP, WBC and eGFR) to explore sources of heterogeneity (Supplementary Table 7). As expected, AF type emerged as an important factor contributing to heterogeneity (*R*^2^ = 55.74%, *p* < 0.0001) in BNP analysis. We conducted subgroup analyses (Supplementary Fig. 6), which showed that studies (six) including only paroxysmal AF (PAF) patients showed that levels of BNP were significantly higher in the recurrence group (OR 2.74, 95% CI: 1.63–4.60, *p* < 0.01, *I*^2^ = 78%, *p* < 0.01). Despite statistical significance, there is a decrease in strength of association of high level of BNP and AF recurrence for studies that included both PAF and persistent AF cohorts [OR 1.95, 95% CI: 1.15–3.31, *p* < 0.05 (*I*^2^ = 85%, *p* < 0.01)]. The addition of long-standing persistent AF populations (3 studies) to PAF and persistent AF showed no statistical difference in BNP levels in the groups of AF recurrence and non-recurrence. Egger’s test (*p* > 0.05) did not illustrate funnel plot asymmetry indicating there was no small-study effect for all the biomarkers that retrieved 10 or more studies (BNP, NT-proBNP, hsCRP, WBC and eGFR; Supplementary Fig. 7).

### Risk of bias in studies

The majority of studies were considered to have a low to moderate (58 articles) risk of bias (Supplementary Table 8). Of the 15 studies classified as having a high risk of bias, none were excluded from the analysis.

## Discussion

**Fig. 6:**
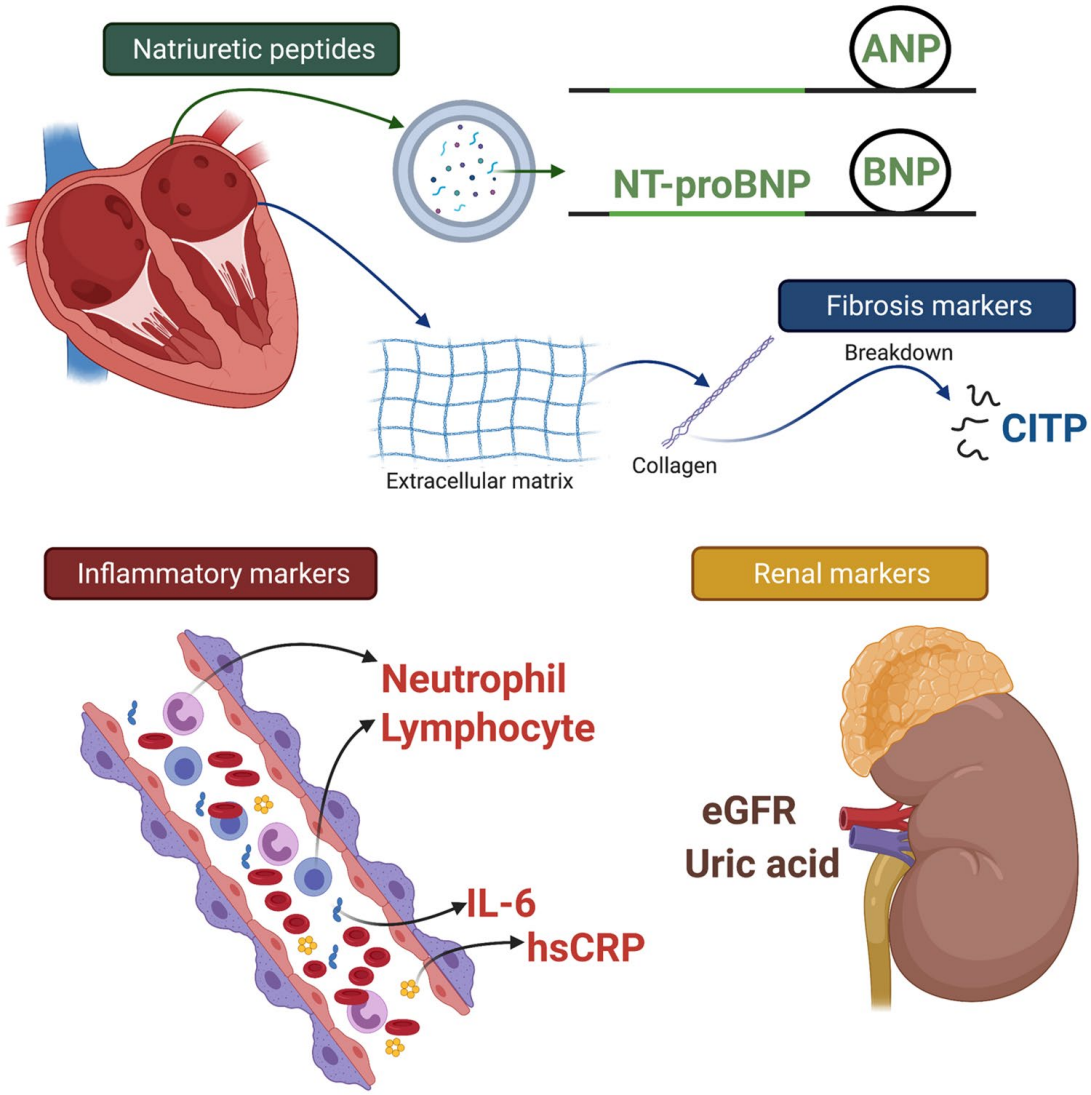
Biomarkers that are significantly associated with recurrence of AF following ablation

This meta-analysis identified 73 studies that investigated baseline biomarkers and their association with AF recurrence following CA. The main findings of this meta-analysis in patients undergoing CA demonstrate: (1) high baseline levels of NT-proBNP, BNP, hsCRP, CITP and IL-6 are strongly associated with recurrence of AF compared to sinus rhythm (2) other biomarkers that were statistically significant but with a lower magnitude of association with AF recurrence include increased baseline levels of ANP, WBC, uric acid and decreased level of eGFR (Fig. 6). AF ablation technology evolved over the last few decades, from improvements in ablation catheters to mapping technologies and evidence-backed ablation strategies [17]. However, in a recent meta-analysis, the risk of arrhythmia recurrence was only reduced by ~ 55% in CA compared to medical therapy [18]. To further improve outcomes, serum biomarkers are positioned to play a role in facilitating personalised medicine as a predictive tool or identify drug targets that can alter biological conditions enabling success.

### Natriuretic peptides

Previous meta-analyses that analysed natriuretic peptides in predicting ablation outcomes possess limitations, i.e. over-estimating effect sizes due to a lack of consistent measurement units and a failure to discriminate natriuretic peptides [12, 19]. Our findings highlight a strong association between elevated B-type natriuretic peptides (BNP, NT-proBNP) and AF recurrence following CA. It has been well-established that BNP and NT-proBNP levels are significantly elevated in AF compared to healthy controls [20–22]. Pathological drivers resulting in raised B-type natriuretic peptides are unique to this disease, highlighted by differences noted in heart failure cohorts with/without AF [23].

The question remains as to whether these drivers are atrial or ventricular in origin? Atrial volume changes in AF are only weak to moderately correlated with BNP levels [24–26]. One possible hypothesis could be that the rise of B-type natriuretic peptides in AF could be the consequence of tachyarrhythmia leading to ventricular dysfunction. In the absence of cardiac hemodynamic changes, inflammation has increased plasma BNP and its gene expression in cardiac tissues [27]. Inflammation is a cause of atrial cardiomyopathy, which has been recently defined as structural and/or electrophysiological changes in the atria, contributing to the development and maintenance of AF [28, 29]. Therefore, elevated natriuretic peptides unique to AF could be driven by inflammation and increase the probability of AF recurrence post-ablation.

Another important finding is that BNP levels were affected by AF type in the subgroup analysis. Non-PAF patients within the cohorts reduced the strength of association of BNP levels with ablation outcomes. In the current literature, higher BNP and NT-proBNP are associated with AF progression [30, 31] and non-PAF [32, 33]. The potential explanation could be that increased BNP is an indicator for a higher arrhythmia burden within PAF cohorts due to frequent paroxysms. These may increase the likelihood of a patient being in AF at the time of the test, consequently elevating the level of B-type natriuretic peptides [34].

### Inflammatory markers

Three previous meta-analyses have combined studies assessing CRP and hsCRP, concluding that CRP is a valuable predictor for recurrence of AF post ablation [10–12]. This meta-analysis is the first to discriminate that hsCRP and not CRP (Supplementary Fig. 2) is associated with ablation outcomes. IL-6 stimulates CRP, and both markers are usually studied together [35]. Histological data assessing left atrial appendages in patients undergoing cardiothoracic surgery showed significantly higher IL-6-positive macrophages in AF patients than controls in sinus rhythm [36]. This indicates that serum IL-6 in patients with AF is a result of local cardiac inflammatory processes. The findings of this meta-analysis strengthen previous studies that have shown an increased risk of AF [37] with IL-6 and its association with recurrence following cardioversion [10] or ablation [12].

### Fibrosis markers

Structural changes in the form of cardiac fibrosis can be characterised by an increase in extracellular matrix deposition [38]. Logically, markers of synthesis and degradation of collagen [Type I and III collagen-related biomarkers (CITP, TIMP, matrix-metalloproteinase (MMP), type III procollagen N-terminal peptide (PIIINP), TGF-b] have been investigated in relation to AF ablation outcomes. A recent study by Ravassa [39] combined fibrosis markers (including CITP), suggesting that the cross-linking and deposition involved in left atrial electrical remodelling is independently predictive of recurrence following CA [39]. This is the first meta-analysis to show pooled effects of CITP and its association with AF ablation outcomes. Modulation of galectin-3 (Gal-3) has contradictory evidence, and modulation does not attenuate cardiac fibrosis [40]. This is in keeping with findings of our meta-analysis that show baseline serum Gal-3 is not associated with AF recurrence following ablation. In contradiction, a recently published meta-analysis demonstrated otherwise, but this finding was probably influenced by a study that collected intra-cardiac blood samples [41]. Begg [42] showed that Gal-3 levels in AF are much higher in peripheral blood compared to intra-cardiac chambers [42], suggesting other potential sources (vascular, renal or hepatic) [40].

### Clinical application

This is the first study to rank serum biomarkers based on their strength of association with AF ablation outcomes. Several prognostic models have been developed for predicting AF recurrence following CA in different regions of the world, and some models include eGFR [43], which is of relatively low priority based on findings in this meta-analysis. The incorporation of natriuretic peptides (BNP and NT-proBNP) [44], fibrosis markers (CITP) [39] or inflammatory marker (hsCRP) to existing prognostic models may improve their performance, especially given their strong association with recurrence. Drugs targeting systemic inflammation (anti-IL-6) may offer to be an alternative approach to anti-arrhythmic therapy given recent findings of IL-6 directly influencing atrial remodelling (down-regulation of atrial connexins) [45, 46]. Better precision medicine models can be achieved if optimised prognostic models identify high-risk groups and utilise drugs to modify their ablation outcomes.

### Limitations

The most important limitation is that majority of the studies included in our analyses were observational (97%). However, prospective studies (67%) made up for a higher proportion. There was also significant heterogeneity in the studies that were utilised for biomarker assessments. The contributing factors to heterogeneity include disproportionate baseline characteristics (more males), variation in sample size (44 to 1410), AF type, clinical management (ablation strategy consisting of additional lines following PVI) and variation in the timing of the outcome measurements (follow-up ranging from 3 to 61 months). It was difficult to ascertain AAD strategy pre-ablation, which could have contributed to variation in the concentration of baseline biomarkers. Despite the exclusion of complex patients (heart failure, structural heart disease, valvular AF), these biomarkers may be easily affected by conditions, such as chronic kidney disease, hepatic failure, respiratory disorders (pulmonary hypertension) or, in some cases, concurrent infection at the time of the blood test. Moreover, there is no consensus for the cut-off values of the biomarkers to predict an outcome.

## Conclusion

Inflammatory markers and natriuretic peptides were shown to have predictive capabilities for AF recurrence in patients undergoing CA. Blood samples are easy to obtain, and processing is relatively inexpensive. Incorporating these biomarkers that are strongly associated with AF recurrence into existing prognostic scores may improve predictive capabilities. Further validation would require a carefully designed RCT to monitor these biomarkers at pre-defined time points across the range of well-characterised patient cohorts (PAF and non-PAF). Subsequently, these biomarkers may finally be incorporated into clinical practice and enable personalisation of both pharmacological and interventional AF therapies.

## Supplementary Information

Below is the link to the electronic supplementary material.

Supplementary file1 (DOCX 136 KB)

Supplementary file2 (DOCX 2626 KB)

Supplementary file3 (DOCX 87 KB)

## Data Availability

Available on reasonable request.

## Code availability

R programme code available on reasonable request.
