# Supplementary material for: Biomarkers as predictors of recurrence of atrial fibrillation post ablation: an updated and expanded systematic review and meta-analysis
Source: Clin Res Cardiol. 2022 Jan 9;111(6):680–91. doi: 10.1007/s00392-021-01978-w (PMC9151522; doi:10.1007/s00392-021-01978-w)
Supplement: Supplementary file 1 — Supplementary file1 (DOCX 136 KB) [file 392_2021_1978_MOESM1_ESM.docx]

Naruse 2011 - BNP

Canpolat 2013

Im 2013 - BNP

Parwani 2015 – NT-proBNP

Clementy 2016

Clementy 2018 - BNP

Deng 2018 - BNP

Deng 2019

Kawaji 2020

| Deng 2018 | Deng H, Shantsila A, Guo P, et al. Multiple biomarkers and arrhythmia outcome following catheter ablation of atrial fibrillation: The Guangzhou Atrial Fibrillation Project. *J Arrhythmia*. 2018;34(6):617-625. | Congestive heart failure in groups – recurrence [n=29 (7.9%)] and non-recurrence [n=42 (4%)] p <0.01.  Although EF 63.0 ± 7.4 65.3 ± 5.5 |
| --- | --- | --- |
| Parwani 2015 | Parwani AS, von Haehling S, Kolodziejski AI, et al. Mid-regional proadrenomedullin levels predict recurrence of atrial fibrillation after catheter ablation. *Int J Cardiol*. 2015;180:129-133. | AF recurrence patients had EF of 50 (10) and non-recurrence group 55 (7) - significant 0.02 |
| Clementy 2016 | Clementy N, Benhenda N, Piver E, et al. Serum Galectin-3 Levels Predict Recurrences after Ablation of Atrial Fibrillation. *Sci Rep*. 2016;6:34357. | No difference in LVEF >40% in groups.  No recurrence -19 (18) recurrence -18 (33) – sig – heart failure0.04 |
| Kawaji 2020 | Kawaji T, Shizuta S, Aizawa T, et al. Renal function and outcomes in atrial fibrillation patients after catheter ablation. Shimosawa T, ed. *PLOS ONE*. 2020;15(11):e0241449. | NR - 37 (7.2%) R-40 (15.1%) - <0.001  LVEF >40% - differecnes |
| Canpolat 2013 | Canpolat U, Aytemir K, Yorgun H, et al. Role of Preablation Neutrophil/Lymphocyte Ratio on Outcomes of Cryoballoon-Based Atrial Fibrillation Ablation. *Am J Cardiol*. 2013;112(4):513-519. | NR - 65.2 + 3.9 R - 62.3 + 9.7 =0.001 |
| Deng 2019 | Deng H, Shantsila A, Guo P, et al. Sex-related risks of recurrence of atrial fibrillation after ablation: Insights from the Guangzhou Atrial Fibrillation Ablation Registry. *Arch Cardiovasc Dis*. 2019;112(3):171-179. | Differences noted between LVEF and CHF history in groups |
| Naruse 2011 | Naruse Y, Tada H, Sekiguchi Y, et al. Concomitant chronic kidney disease increases the recurrence of atrial fibrillation after catheter ablation of atrial fibrillation: A mid-term follow-up. *Heart Rhythm*. 2011;8(3):335-341. | LVEF in both groups >50%, difference is signifncant |
| Clementy 2018 | Clementy N, Garcia B, André C, et al. Galectin-3 level predicts response to ablation and outcomes in patients with persistent atrial fibrillation and systolic heart failure. *PloS One*. 2018;13(8):e0201517. | No difference between groups – but need to consider removing as patients are systolic heart failure |
| Im 2013 | Im SI, Shin SY, Na JO, et al. Usefulness of neutrophil/lymphocyte ratio in predicting early recurrence after radiofrequency catheter ablation in patients with atrial fibrillation. *Int J Cardiol*. 2013;168(4):4398-4400. | Differences between CHF and LVEF |

| Article | Heart failure/LVEF |
| --- | --- |
| Deng H, Shantsila A, Guo P, et al. Multiple biomarkers and arrhythmia outcome following catheter ablation of atrial fibrillation: The Guangzhou Atrial Fibrillation Project. *J Arrhythmia*. 2018;34(6):617-625. | Congestive heart failure in groups – recurrence [n=29 (7.9%)] and non-recurrence [n=42 (4%)] p <0.01.  Although EF 63.0 ± 7.4 65.3 ± 5.5 |
| Okumura Y, Watanabe I, Nakai T, et al. Impact of biomarkers of inflammation and extracellular matrix turnover on the outcome of atrial fibrillation ablation: importance of matrix metalloproteinase-2 as a predictor of atrial fibrillation recurrence. *J Cardiovasc Electrophysiol*. 2011;22(9):987-993. | Heart failure total number in non recurrence is 1 (3.5%) and in recurrence is 2 (9.5%) |
| Pillarisetti J, Reddy N, Biria M, et al. Elevated brain natriuretic peptide level in patients undergoing atrial fibrillation ablation: is it a predictor of failed ablation or a mere function of atrial rhythm and rate at a point in time? *J Interv Card Electrophysiol Int J Arrhythm Pacing*. 2014;40(2):161-168. | EF for the whole group 57±8 |
| Kimura T, Takatsuki S, Inagawa K, et al. Serum inflammation markers predicting successful initial catheter ablation for atrial fibrillation. *Heart Lung Circ*. 2014;23(7):636-643. | No differences in LVEF between groups (72.7 + 9.0 vs 70.5 + 10.1, p= 0.456). |
| Tamura S, Doi A, Matsuo M, et al. Prognostic value of high‐sensitive troponin T for predicting cardiovascular events after atrial fibrillation ablation. *J Cardiovasc Electrophysiol*. 2019;30(9):1475-1482. | EF in recurrence 56 ± 9 vs non-recurrence 57 ± 8, non-sginficant |
| Huang Z, Liang X, Wang W, et al. Relationship between plasma cancer antigen (CA)-125 level and one-year recurrence of atrial fibrillation after catheter ablation. *Clin Chim ACTA*. 2020;502:201-206 | EF 64.72 +7.68 in non-recurrence and 62.53 + 10.75 in recurrence, NS |
| den Uijl DW, Delgado V, Tops LF, et al. Natriuretic peptide levels predict recurrence of atrial fibrillation after radiofrequency catheter ablation. *Am Heart J*. 2011;161(1):197-203. | EF in non-recurrence 59 ± 6 vs recurrence group 57 ± 4 .32 - NS |
| Parwani AS, von Haehling S, Kolodziejski AI, et al. Mid-regional proadrenomedullin levels predict recurrence of atrial fibrillation after catheter ablation. *Int J Cardiol*. 2015;180:129-133. | AF recurrence patients had EF of 50 (10) and non-recurrence group 55 (7) - significant 0.02 |
| Liu L, Zhao D, Zhang J, et al. Impact of Stable Coronary Artery Disease on the Efficacy of Cryoballoon Ablation for the Atrial Fibrillation. *Am J Med Sci*. 2019;358(3):204-211. | AF non-recurrence 59.3 +8.3 and recurrence group 63.9 + 4.1 – NS  LVEF in both groups >50%, difference is NS |
| Du W, Dai M, Wang M, et al. Large left atrial appendage predicts the ablation outcome in hypertensive patients with atrial fibrillation. *J Electrocardiol*. 2020;63:139-144. | No recurrence 65.79 ± 4.29 vs recurrence 65.68 ± 2.88 - NS  LVEF in both groups >60%, difference is NS |
| Can V, Cakmak HA, Vatansever F, et al. Assessment of the relationship between semaphorin4D level and recurrence after catheter ablation in paroxysmal atrial fibrillation. *Biomarkers*. Published online April 28, 2021:1-9. | LVEF in both groups >50%, difference is NS |
| Letsas KP, Weber R, Bürkle G, et al. Pre-ablative predictors of atrial fibrillation recurrence following pulmonary vein isolation: the potential role of inflammation. *Eur Eur Pacing Arrhythm Card Electrophysiol J Work Groups Card Pacing Arrhythm Card Cell Electrophysiol Eur Soc Cardiol*. 2009;11(2):158-163. | R 54.9 + 10.2 NR 59.6 + 7.4 - S |
| He X, Li S, Zhan J, et al. Serum uric acid levels correlate with recurrence of paroxysmal atrial fibrillation after catheter ablation. *Chin Med J (Engl)*. 2013;126(5):860-864 | na |
| Guo X, Zhang S, Yan X, et al. Postablation neutrophil/lymphocyte ratio correlates with arrhythmia recurrence after catheter ablation of lone atrial fibrillation. *Chin Med J (Engl)*. 2014;127(6):1033-1038. | LVEF in both groups >50%, difference is NS |
| Aksu T, Baysal E, Guler TE, Golcuk SE, Erden İ, Ozcan KS. Predictors of atrial fibrillation recurrence after cryoballoon ablation. *J Blood Med*. 2015;6:211-217. | LVEF in both groups >50%, difference is NS |
| Chen S, Zhang M, Zheng M, et al. The preablation monocyte/high density lipoprotein ratio predicts the late recurrence of paroxysmal atrial fibrillation after radiofrequency ablation. *BMC Cardiovasc Disord*. 2020;20(1). | LVEF in both groups >55%, difference is NS |
| Ravassa S, Ballesteros G, López B, et al. Combination of Circulating Type I Collagen-Related Biomarkers Is Associated With Atrial Fibrillation. *J Am Coll Cardiol*. 2019;73(12):1398-1410. | LVEF in both groups >50%, difference is NS  No difference in heart failure patients in groups |
| Clementy N, Benhenda N, Piver E, et al. Serum Galectin-3 Levels Predict Recurrences after Ablation of Atrial Fibrillation. *Sci Rep*. 2016;6:34357. | No difference in LVEF >40% in groups.  No recurrence -19 (18) recurrence -18 (33) – sig – heart failure0.04 |
| Macías-Ruiz R, Jiménez-Jáimez J, Álvarez-López M, et al. Effect of pulmonary vein catheter ablation on kidney function in patients with atrial fibrillation. A prospective cohort study. *Rev Esp Cardiol Engl Ed*. 2020;73(6):471-478. | NA |
| Kawaji T, Shizuta S, Aizawa T, et al. Renal function and outcomes in atrial fibrillation patients after catheter ablation. Shimosawa T, ed. *PLOS ONE*. 2020;15(11):e0241449. | NR - 37 (7.2%) R-40 (15.1%) - <0.001  LVEF >40% - differecnes |
| Canpolat U, Aytemir K, Yorgun H, et al. Usefulness of serum uric acid level to predict atrial fibrillation recurrence after cryoballoon-based catheter ablation. *Eur Eur Pacing Arrhythm Card Electrophysiol J Work Groups Card Pacing Arrhythm Card Cell Electrophysiol Eur Soc Cardiol*. 2014;16(12):1731-1737. | LVEF in both groups >60%, difference is NS |
| Celik AI, Kanadasi M, Demir M, et al. Predictors of the paroxysmal atrial fibrillation recurrence following cryoballoon-based pulmonary vein isolation: Assessment of left atrial volume, left atrial volume index, galectin-3 level and neutrophil-to-lymphocyte ratio. *Indian Pacing Electrophysiol J*. 2019;19(1):9-14. | NA |
| Canpolat U, Aytemir K, Yorgun H, et al. Role of Preablation Neutrophil/Lymphocyte Ratio on Outcomes of Cryoballoon-Based Atrial Fibrillation Ablation. *Am J Cardiol*. 2013;112(4):513-519. | NR - 65.2 + 3.9 R - 62.3 + 9.7 =0.001 |
| Aksu T, Golcuk SE, Guler TE, Yalin K, Erden I. Prediction of mid-term outcome after cryo-balloon ablation of atrial fibrillation using post-procedure high-sensitivity troponin level. *Cardiovasc J Afr*. 2015;26(4):165-170. | LVEF in both groups >50%, difference is NS |
| Date T, Yamane T, Inada K, et al. Plasma brain natriuretic peptide concentrations in patients undergoing pulmonary vein isolation. *Heart*. 2006;92(11):1623-1627. | NA |
| Machino-Ohtsuka T, Seo Y, Tada H, et al. Left Atrial Stiffness Relates to Left Ventricular Diastolic Dysfunction and Recurrence After Pulmonary Vein Isolation for Atrial Fibrillation. *J Cardiovasc Electrophysiol*. 2011;22(9):999-1006. | NA – EF not predictor of success |
| Yamada T, Murakami Y, Okada T, et al. Electrophysiological pulmonary vein antrum isolation with a multielectrode basket catheter is feasible and effective for curing paroxysmal atrial fibrillation: efficacy of minimally extensive pulmonary vein isolation. *Heart Rhythm*. 2006;3(4):377-384. | NA |
| Shaikh AY, Esa N, Martin-Doyle W, et al. Addition of B-Type Natriuretic Peptide to Existing Clinical Risk Scores Enhances Identification of Patients at Risk for Atrial Fibrillation Recurrence After Pulmonary Vein Isolation. *Crit Pathw Cardiol*. 2015;14(4):157-165. | No difference in CHF in groups |
| Kishima H, Mine T, Takahashi S, Ashida K, Ishihara M, Masuyama T. The Impact of Transforming Growth Factor-β1 Level on Outcome After Catheter Ablation in Patients With Atrial Fibrillation. *J Cardiovasc Electrophysiol*. 2017;28(4):402-409. | LVEF in both groups >50%, difference is NS |
| Huang Q, Yuan Y, Qiu C, et al. Effect of catheter radiofrequency ablation on C-reactive protein, brain natriuretic peptide and echocardiograph in patients with persistent and permanent atrial fibrillation. *Chin Med J (Engl)*. 2014;127(4):623-626. | LVEF in both groups >50%, difference is NS |
| Xu M, Liu F, Ge Z-X, Li J-M, Xie X, Yang J-H. Functional studies of left atrium and BNP in patients with paroxysmal atrial fibrillation and the prediction of recurrence after CPVA. *Eur Rev Med Pharmacol Sci*. 2020;24(9):4997-5007. | LVEF in both groups >50%, difference is NS  (((((31) |
| Yano M, Egami Y, Ukita K, et al. Atrial fibrillation type modulates the clinical predictive value of neutrophil-to-lymphocyte ratio for atrial fibrillation recurrence after catheter ablation. *IJC Heart Vasc*. 2020;31:100664. | LVEF in both groups >60%, difference is NS |
| Nilsson B, Goetze JP, Chen X, Pehrson S, Svendsen JH. Increased NT-pro-B-type natriuretic peptide independently predicts outcome following catheter ablation of atrial fibrillation. *Scand J Clin Lab Invest*. 2009;69(8):843-850. | NA |
| Su C, Liu Z, Gao Y, et al. Study on the relationship between telomere length changes and recurrence of atrial fibrillation after radiofrequency catheter ablation. *J Cardiovasc Electrophysiol*. Published online May 1, 2019. | LV dysfunction and LVEF no difference between groups. LVEF >55% in both groups |
| Ma X-X, Zhang Y-L, Hu B, et al. Association between left atrial appendage emptying velocity, N-terminal plasma brain natriuretic peptide levels, and recurrence of atrial fibrillation after catheter ablation. *J Interv Card Electrophysiol Int J Arrhythm Pacing*. 2017;48(3):343-350. | LVEF in both groups >50%, difference is NS |
| Liu H, Wang K, Lin Y, et al. Role of sST2 in predicting recurrence of atrial fibrillation after radiofrequency catheter ablation. *PACE-PACING Clin Electrophysiol*. 2020;43(11):1235-1241. | LVEF in both groups >55%, difference is NS |
| Henningsen KMA, Therkelsen SK, Bruunsgaard H, Krabbe KS, Pedersen BK, Svendsen JH. Prognostic impact of hs-CRP and IL-6 in patients with persistent atrial fibrillation treated with electrical cardioversion. *Scand J Clin Lab Invest*. 2009;69(3):425-432. | LVEF in both groups >50%, difference is NS |
| Shin SY, Na JO, Lim HE, et al. Improved endothelial function in patients with atrial fibrillation through maintenance of sinus rhythm by successful catheter ablation. *J Cardiovasc Electrophysiol*. 2011;22(4):376-382. | LVEF in both groups >50%, difference is NS ((((38) |
| Kornej J, Reinhardt C, Kosiuk J, et al. Response of circulating heat shock protein 70 and anti-heat shock protein 70 antibodies to catheter ablation of atrial fibrillation. *J Transl Med*. 2013;11:49. | LVEF in both groups >45%, difference is NS |
| Wang H, Liu J, Fang P, et al. Big endothelin-1 as a predictor of atrial fibrillation recurrence after primary ablation only in patients with paroxysmal atrial fibrillation. *Herz*. 2012;37(8):919-925. | LVEF in both groups >50%, difference is NS |
| Bazoukis G, Letsas KP, Vlachos K, et al. Simple hematological predictors of AF recurrence in patients undergoing atrial fibrillation ablation. *J Geriatr Cardiol JGC*. 2019;16(9):671-675 | NA |
| Cabrera-Bueno F, Medina-Palomo C, Ruiz-Salas A, et al. Serum levels of interleukin-2 predict the recurrence of atrial fibrillation after pulmonary vein ablation. *Cytokine*. 2015;73(1):74-78. | LVEF in both groups >60%, difference is NS |
| Qu X, Chen L, Sun L, et al. Serum relaxin level predicts recurrence of atrial fibrillation after radiofrequency catheter ablation. *Heart Vessels*. 2019;34(9):1543-1551. | LVEF in both groups >50%, difference is NS |
| Miake J, Kato M, Ogura K, et al. Pre-ablation levels of brain natriuretic peptide are independently associated with the recurrence of atrial fibrillation after radiofrequency catheter ablation in patients with nonvalvular atrial fibrillation. *Heart Vessels*. 2019;34(3):517-526. |  |
| Tokuda M, Yamane T, Matsuo S, et al. Relationship between renal function and the risk of recurrent atrial fibrillation following catheter ablation. *Heart Br Card Soc*. 2011;97(2):137-142. |  |
| Li A, Chen Y, Wang W, Su L, Ling Z. Association of clinical predictors with recurrence of atrial fibrillation after catheter ablation. *Ann Noninvasive Electrocardiol*. 2020;25(6). |  |
| Deng H, Shantsila A, Guo P, et al. Sex-related risks of recurrence of atrial fibrillation after ablation: Insights from the Guangzhou Atrial Fibrillation Ablation Registry. *Arch Cardiovasc Dis*. 2019;112(3):171-179. | Differences noted between LVEF and CHF history in groups |
| Wu C-H, Hu Y-F, Chou C-Y, et al. Transforming growth factor-β1 level and outcome after catheter ablation for nonparoxysmal atrial fibrillation. *Heart Rhythm*. 2013;10(1):10-15. | LVEF in both groups >40%, difference is NS |
| Yano M, Egami Y, Yanagawa K, et al. Predictors of recurrence after pulmonary vein isolation in patients with normal left atrial diameter. *J Arrhythmia*. 2020;36(1):75-81. | LVEF in both groups >60%, difference is NS and no difference between CHF in groups. |
| Naruse Y, Tada H, Sekiguchi Y, et al. Concomitant chronic kidney disease increases the recurrence of atrial fibrillation after catheter ablation of atrial fibrillation: A mid-term follow-up. *Heart Rhythm*. 2011;8(3):335-341. | LVEF in both groups >50%, difference is signifncant |
| Clementy N, Garcia B, André C, et al. Galectin-3 level predicts response to ablation and outcomes in patients with persistent atrial fibrillation and systolic heart failure. *PloS One*. 2018;13(8):e0201517. | No difference between groups – but need to consider removing as patients are systolic heart failure |
| Yanagisawa S, Inden Y, Kato H, et al. Elevated Red Blood Cell Distribution Width Predicts Recurrence After Catheter Ablation for Atrial Fibrillation in Patients With Heart Failure　- Comparison With Non-Heart Failure Patients. *Circ J Off J Jpn Circ Soc*. 2016;80(3):627-638. | Used non-HF group results, therefore no differences between LVEF in groups. |
| Nakazawa Y, Ashihara T, Tsutamoto T, Ito M, Horie M. Endothelin-1 as a predictor of atrial fibrillation recurrence after pulmonary vein isolation. *Heart Rhythm*. 2009;6(6):725-730. | LVEF in both groups >50%, difference is NS |
| Im SI, Shin SY, Na JO, et al. Usefulness of neutrophil/lymphocyte ratio in predicting early recurrence after radiofrequency catheter ablation in patients with atrial fibrillation. *Int J Cardiol*. 2013;168(4):4398-4400. | Differences between CHF and LVEF |
| Wu X-Y, Li S-N, Wen S-N, et al. Plasma galectin-3 predicts clinical outcomes after catheter ablation in persistent atrial fibrillation patients without structural heart disease. *Eur Eur Pacing Arrhythm Card Electrophysiol J Work Groups Card Pacing Arrhythm Card Cell Electrophysiol Eur Soc Cardiol*. 2015;17(10):1541-1547. | LVEF in both groups >55%, difference is NS |
| Oka T, Tanaka K, Ninomiya Y, et al. Impact of baseline left atrial function on long-term outcome after catheter ablation for paroxysmal atrial fibrillation. *J Cardiol*. 2020;75(4):352-359. | LVEF in both groups >60%, difference is NS and no difference in CHF groups |
| Luetkens JA, Wolpers AC, Beiert T, et al. Cardiac magnetic resonance using late gadolinium enhancement and atrial T1 mapping predicts poor outcome in patients with atrial fibrillation after catheter ablation therapy. *Sci Rep*. 2018;8(1):13618. | NA |
| Shiozawa T, Shimada K, Sekita G, et al. Left Atrial Appendage Volume and Plasma Docosahexaenoic Acid Levels Are Associated With Atrial Fibrillation Recurrence After Catheter Ablation. *Cardiol Res*. 2017;8(3):96-104. | LVEF in both groups >55%, difference is NS |
| Zou C, Zhang Z, Zhao W, et al. Predictive value of pre-procedural autoantibodies against M2-muscarinic acetylcholine receptor for recurrence of atrial fibrillation one year after radiofrequency catheter ablation. *J Transl Med*. 2013;11(1):7. | LVEF in both groups >60%, difference is NS |
| Nakamura K, Takagi T, Kogame N, et al. Impact of atrial mitral and tricuspid regurgitation on atrial fibrillation recurrence after ablation. *J Electrocardiol*. 2021;66:114-121. | No difference in heart failure or LVEF >45% |
| Wei Y, Liu S, Yu H, et al. The Predictive Value of Growth Differentiation Factor-15 in Recurrence of Atrial Fibrillation after Catheter Ablation. *Mediators Inflamm*. 2020;2020. | No difference in heart failure but difference in LVEF – despite being >64% in both groups |
| Liu J, Fang P-H, Dibs S, Hou Y, Li X-F, Zhang S. High-Sensitivity C-Reactive Protein as a Predictor of Atrial Fibrillation Recurrence after Primary Circumferential Pulmonary Vein Isolation: ATRIAL FIBRILLATION ABLATION RECURRENCE hsCRP. *Pacing Clin Electrophysiol*. 2011;34(4):398-406. | No difference in LVEF |
| Li S, Yang F, Jing L, et al. Myeloperoxidase and risk of recurrence of atrial fibrillation after catheter ablation. *J Investig Med Off Publ Am Fed Clin Res*. 2013;61(4):722-727. | LVEF in both groups >50%, difference is NS |
| Namino F, Yamakuchi M, Iriki Y, et al. Dynamics of Soluble Thrombomodulin and Circulating miRNAs in Patients with Atrial Fibrillation Undergoing Radiofrequency Catheter Ablation. *Clin Appl Thromb*. 2019;25:107602961985157. | No difference in heart failure or LVEF |
| Canpolat U, Aytemir K, Yorgun H, et al. The role of preprocedural monocyte-to-high-density lipoprotein ratio in prediction of atrial fibrillation recurrence after cryoballoon-based catheter ablation. *Eur Eur Pacing Arrhythm Card Electrophysiol J Work Groups Card Pacing Arrhythm Card Cell Electrophysiol Eur Soc Cardiol*. 2015;17(12):1807-1815. | No difference in LVEF >60% |
| Shim J, Park JH, Kim JY, et al. Impaired mobilization of bone marrow derived CD34 positive mononuclear cells is related to the recurrence of atrial fibrillation after radiofrequency catheter ablation. *Int J Cardiol*. 2013;162(3):179-183. | No difference in LVEF >45% |
| Kornej J, Schmidl J, Ueberham L, et al. Galectin-3 in patients with atrial fibrillation undergoing radiofrequency catheter ablation. *PloS One*. 2015;10(4):e0123574. | No difference in LVEF >50% |
| Uçar FM, Gucuk İpek E, Acar B, et al. Gamma-glutamyl transferase predicts recurrences of atrial fibrillation after catheter ablation. *Acta Cardiol*. 2016;71(2):205-210. | NA |
| Okawa K, Miyoshi T, Sogo M, et al. Improvement in renal and endothelial function after catheter ablation in patients with persistent atrial fibrillation. *J Cardiol*. 2020;76(6):610-617. | NA |
| Lellouche N, Sacher F, Wright M, et al. Usefulness of C-reactive protein in predicting early and late recurrences after atrial fibrillation ablation. *Europace*. 2009;11(5):662-664. | LVEF in both groups >50%, difference is NS |
| Choi J-I, Baek YS, Roh SY, Piccini JP, Kim Y-H. Chromosome 4q25 variants and biomarkers of myocardial fibrosis in patients with atrial fibrillation. *J Cardiovasc Electrophysiol*. 2019;30(10):1904-1913. | NA |
| Gunes HM, Babur Guler G, Guler E, et al. Relationship between serum osteopontin level and atrial fibrillation recurrence in patients undergoing cryoballoon catheter ablation. *Turk Kardiyol Dernegi Arsivi Turk Kardiyol Derneginin Yayin Organidir*. 2017;45(1):26-32. | LVEF in both groups >60%, difference is NS |

**Supplementary references**

List of biomarkers that were statistically significant for outcome

1. ANP^1–5^
2. BNP^3–21^
3. NT-proBNP^22–32,33(p2),34,35^
4. hsCRP^4,7,12,14–17,26,27,34–45^
5. WBC^12,21,23,35,37,38,40,41,43–49^
6. IL-6^4,23,36,39,50,51^
7. CITP^1,4,8^
8. eGFR^7,9,12,20,32,35,40,43,52–62^
9. Uric acid^35,40,43,49,62,63^

List of non-significant biomarkers for outcome:

1. CRP^1,8,9,11,20,21,25,47,55,64,65^
2. Gal-3^17,53,54,66^
3. TNF^1,50,51^
4. TIMP^1,4,67^
5. TGF-β^1,2,67,68^
6. NLR^14,21,34,43,47,48,66,69^
7. Cholesterol^12,14,28,31,34,40,41,43^
8. LDL^1,14,26–28,31,34,40,41,43,44,49,57,62^
9. HDL^14,26,31,34,41,43,44,49,62^
10. TG^14,31,34,40,41,43,44^
11. Cr^1,9,12,18,21,23,25,26,29,31,31,32,35,41,44,45,48,49,55,62,70,71^
12. Troponin I^50,64,72^
13. HbA1c^1,26,35^

**References**

1. Kimura T, Takatsuki S, Inagawa K, et al. Serum inflammation markers predicting successful initial catheter ablation for atrial fibrillation. *Heart Lung Circ*. 2014;23(7):636-643.

2. Kishima H, Mine T, Takahashi S, Ashida K, Ishihara M, Masuyama T. The Impact of Transforming Growth Factor-β1 Level on Outcome After Catheter Ablation in Patients With Atrial Fibrillation. *J Cardiovasc Electrophysiol*. 2017;28(4):402-409.

3. Nakazawa Y, Ashihara T, Tsutamoto T, Ito M, Horie M. Endothelin-1 as a predictor of atrial fibrillation recurrence after pulmonary vein isolation. *Heart Rhythm*. 2009;6(6):725-730.

4. Okumura Y, Watanabe I, Nakai T, et al. Impact of biomarkers of inflammation and extracellular matrix turnover on the outcome of atrial fibrillation ablation: importance of matrix metalloproteinase-2 as a predictor of atrial fibrillation recurrence. *J Cardiovasc Electrophysiol*. 2011;22(9):987-993.

5. Yamada T, Murakami Y, Okada T, et al. Electrophysiological pulmonary vein antrum isolation with a multielectrode basket catheter is feasible and effective for curing paroxysmal atrial fibrillation: efficacy of minimally extensive pulmonary vein isolation. *Heart Rhythm*. 2006;3(4):377-384.

6. Clementy N, Garcia B, André C, et al. Galectin-3 level predicts response to ablation and outcomes in patients with persistent atrial fibrillation and systolic heart failure. *PloS One*. 2018;13(8):e0201517.

7. Deng H, Shantsila A, Guo P, et al. Multiple biomarkers and arrhythmia outcome following catheter ablation of atrial fibrillation: The Guangzhou Atrial Fibrillation Project. *J Arrhythmia*. 2018;34(6):617-625.

8. Machino-Ohtsuka T, Seo Y, Tada H, et al. Left Atrial Stiffness Relates to Left Ventricular Diastolic Dysfunction and Recurrence After Pulmonary Vein Isolation for Atrial Fibrillation. *J Cardiovasc Electrophysiol*. 2011;22(9):999-1006.

9. Naruse Y, Tada H, Sekiguchi Y, et al. Concomitant chronic kidney disease increases the recurrence of atrial fibrillation after catheter ablation of atrial fibrillation: A mid-term follow-up. *Heart Rhythm*. 2011;8(3):335-341.

10. Pillarisetti J, Reddy N, Biria M, et al. Elevated brain natriuretic peptide level in patients undergoing atrial fibrillation ablation: is it a predictor of failed ablation or a mere function of atrial rhythm and rate at a point in time? *J Interv Card Electrophysiol Int J Arrhythm Pacing*. 2014;40(2):161-168.

11. Shaikh AY, Esa N, Martin-Doyle W, et al. Addition of B-Type Natriuretic Peptide to Existing Clinical Risk Scores Enhances Identification of Patients at Risk for Atrial Fibrillation Recurrence After Pulmonary Vein Isolation. *Crit Pathw Cardiol*. 2015;14(4):157-165.

12. Yanagisawa S, Inden Y, Kato H, et al. Elevated Red Blood Cell Distribution Width Predicts Recurrence After Catheter Ablation for Atrial Fibrillation in Patients With Heart Failure　- Comparison With Non-Heart Failure Patients. *Circ J Off J Jpn Circ Soc*. 2016;80(3):627-638.

13. Date T, Yamane T, Inada K, et al. Plasma brain natriuretic peptide concentrations in patients undergoing pulmonary vein isolation. *Heart*. 2006;92(11):1623-1627.

14. Im SI, Shin SY, Na JO, et al. Usefulness of neutrophil/lymphocyte ratio in predicting early recurrence after radiofrequency catheter ablation in patients with atrial fibrillation. *Int J Cardiol*. 2013;168(4):4398-4400.

15. Huang Q, Yuan Y, Qiu C, et al. Effect of catheter radiofrequency ablation on C-reactive protein, brain natriuretic peptide and echocardiograph in patients with persistent and permanent atrial fibrillation. *Chin Med J (Engl)*. 2014;127(4):623-626.

16. Tamura S, Doi A, Matsuo M, et al. Prognostic value of high‐sensitive troponin T for predicting cardiovascular events after atrial fibrillation ablation. *J Cardiovasc Electrophysiol*. 2019;30(9):1475-1482.

17. Wu X-Y, Li S-N, Wen S-N, et al. Plasma galectin-3 predicts clinical outcomes after catheter ablation in persistent atrial fibrillation patients without structural heart disease. *Eur Eur Pacing Arrhythm Card Electrophysiol J Work Groups Card Pacing Arrhythm Card Cell Electrophysiol Eur Soc Cardiol*. 2015;17(10):1541-1547.

18. Xu M, Liu F, Ge Z-X, Li J-M, Xie X, Yang J-H. Functional studies of left atrium and BNP in patients with paroxysmal atrial fibrillation and the prediction of recurrence after CPVA. *Eur Rev Med Pharmacol Sci*. 2020;24(9):4997-5007.

19. Huang Z, Liang X, Wang W, et al. Relationship between plasma cancer antigen (CA)-125 level and one-year recurrence of atrial fibrillation after catheter ablation. *Clin Chim ACTA*. 2020;502:201-206.

20. Oka T, Tanaka K, Ninomiya Y, et al. Impact of baseline left atrial function on long-term outcome after catheter ablation for paroxysmal atrial fibrillation. *J Cardiol*. 2020;75(4):352-359.

21. Yano M, Egami Y, Ukita K, et al. Atrial fibrillation type modulates the clinical predictive value of neutrophil-to-lymphocyte ratio for atrial fibrillation recurrence after catheter ablation. *IJC Heart Vasc*. 2020;31:100664.

22. den Uijl DW, Delgado V, Tops LF, et al. Natriuretic peptide levels predict recurrence of atrial fibrillation after radiofrequency catheter ablation. *Am Heart J*. 2011;161(1):197-203.

23. Luetkens JA, Wolpers AC, Beiert T, et al. Cardiac magnetic resonance using late gadolinium enhancement and atrial T1 mapping predicts poor outcome in patients with atrial fibrillation after catheter ablation therapy. *Sci Rep*. 2018;8(1):13618.

24. Nilsson B, Goetze JP, Chen X, Pehrson S, Svendsen JH. Increased NT-pro-B-type natriuretic peptide independently predicts outcome following catheter ablation of atrial fibrillation. *Scand J Clin Lab Invest*. 2009;69(8):843-850.

25. Parwani AS, von Haehling S, Kolodziejski AI, et al. Mid-regional proadrenomedullin levels predict recurrence of atrial fibrillation after catheter ablation. *Int J Cardiol*. 2015;180:129-133.

26. Shiozawa T, Shimada K, Sekita G, et al. Left Atrial Appendage Volume and Plasma Docosahexaenoic Acid Levels Are Associated With Atrial Fibrillation Recurrence After Catheter Ablation. *Cardiol Res*. 2017;8(3):96-104.

27. Su C, Liu Z, Gao Y, et al. Study on the relationship between telomere length changes and recurrence of atrial fibrillation after radiofrequency catheter ablation. *J Cardiovasc Electrophysiol*. Published online May 1, 2019.

28. Liu L, Zhao D, Zhang J, et al. Impact of Stable Coronary Artery Disease on the Efficacy of Cryoballoon Ablation for the Atrial Fibrillation. *Am J Med Sci*. 2019;358(3):204-211.

29. Zou C, Zhang Z, Zhao W, et al. Predictive value of pre-procedural autoantibodies against M2-muscarinic acetylcholine receptor for recurrence of atrial fibrillation one year after radiofrequency catheter ablation. *J Transl Med*. 2013;11(1):7.

30. Ma X-X, Zhang Y-L, Hu B, et al. Association between left atrial appendage emptying velocity, N-terminal plasma brain natriuretic peptide levels, and recurrence of atrial fibrillation after catheter ablation. *J Interv Card Electrophysiol Int J Arrhythm Pacing*. 2017;48(3):343-350.

31. Du W, Dai M, Wang M, et al. Large left atrial appendage predicts the ablation outcome in hypertensive patients with atrial fibrillation. *J Electrocardiol*. 2020;63:139-144.

32. Nakamura K, Takagi T, Kogame N, et al. Impact of atrial mitral and tricuspid regurgitation on atrial fibrillation recurrence after ablation. *J Electrocardiol*. 2021;66:114-121.

33. Liu H, Wang K, Lin Y, et al. Role of sST2 in predicting recurrence of atrial fibrillation after radiofrequency catheter ablation. *PACE-PACING Clin Electrophysiol*. 2020;43(11):1235-1241.

34. Can V, Cakmak HA, Vatansever F, et al. Assessment of the relationship between semaphorin4D level and recurrence after catheter ablation in paroxysmal atrial fibrillation. *Biomarkers*. Published online April 28, 2021:1-9.

35. Wei Y, Liu S, Yu H, et al. The Predictive Value of Growth Differentiation Factor-15 in Recurrence of Atrial Fibrillation after Catheter Ablation. *Mediators Inflamm*. 2020;2020.

36. Henningsen KMA, Therkelsen SK, Bruunsgaard H, Krabbe KS, Pedersen BK, Svendsen JH. Prognostic impact of hs-CRP and IL-6 in patients with persistent atrial fibrillation treated with electrical cardioversion. *Scand J Clin Lab Invest*. 2009;69(3):425-432.

37. Letsas KP, Weber R, Bürkle G, et al. Pre-ablative predictors of atrial fibrillation recurrence following pulmonary vein isolation: the potential role of inflammation. *Eur Eur Pacing Arrhythm Card Electrophysiol J Work Groups Card Pacing Arrhythm Card Cell Electrophysiol Eur Soc Cardiol*. 2009;11(2):158-163.

38. Liu J, Fang P-H, Dibs S, Hou Y, Li X-F, Zhang S. High-Sensitivity C-Reactive Protein as a Predictor of Atrial Fibrillation Recurrence after Primary Circumferential Pulmonary Vein Isolation: ATRIAL FIBRILLATION ABLATION RECURRENCE hsCRP. *Pacing Clin Electrophysiol*. 2011;34(4):398-406.

39. Shin SY, Na JO, Lim HE, et al. Improved endothelial function in patients with atrial fibrillation through maintenance of sinus rhythm by successful catheter ablation. *J Cardiovasc Electrophysiol*. 2011;22(4):376-382.

40. He X, Li S, Zhan J, et al. Serum uric acid levels correlate with recurrence of paroxysmal atrial fibrillation after catheter ablation. *Chin Med J (Engl)*. 2013;126(5):860-864.

41. Li S, Yang F, Jing L, et al. Myeloperoxidase and risk of recurrence of atrial fibrillation after catheter ablation. *J Investig Med Off Publ Am Fed Clin Res*. 2013;61(4):722-727.

42. Kornej J, Reinhardt C, Kosiuk J, et al. Response of circulating heat shock protein 70 and anti-heat shock protein 70 antibodies to catheter ablation of atrial fibrillation. *J Transl Med*. 2013;11:49.

43. Guo X, Zhang S, Yan X, et al. Postablation neutrophil/lymphocyte ratio correlates with arrhythmia recurrence after catheter ablation of lone atrial fibrillation. *Chin Med J (Engl)*. 2014;127(6):1033-1038.

44. Canpolat U, Aytemir K, Yorgun H, et al. The role of preprocedural monocyte-to-high-density lipoprotein ratio in prediction of atrial fibrillation recurrence after cryoballoon-based catheter ablation. *Eur Eur Pacing Arrhythm Card Electrophysiol J Work Groups Card Pacing Arrhythm Card Cell Electrophysiol Eur Soc Cardiol*. 2015;17(12):1807-1815.

45. Namino F, Yamakuchi M, Iriki Y, et al. Dynamics of Soluble Thrombomodulin and Circulating miRNAs in Patients with Atrial Fibrillation Undergoing Radiofrequency Catheter Ablation. *Clin Appl Thromb*. 2019;25:107602961985157.

46. Wang H, Liu J, Fang P, et al. Big endothelin-1 as a predictor of atrial fibrillation recurrence after primary ablation only in patients with paroxysmal atrial fibrillation. *Herz*. 2012;37(8):919-925.

47. Aksu T, Baysal E, Guler TE, Golcuk SE, Erden İ, Ozcan KS. Predictors of atrial fibrillation recurrence after cryoballoon ablation. *J Blood Med*. 2015;6:211-217.

48. Bazoukis G, Letsas KP, Vlachos K, et al. Simple hematological predictors of AF recurrence in patients undergoing atrial fibrillation ablation. *J Geriatr Cardiol JGC*. 2019;16(9):671-675.

49. Chen S, Zhang M, Zheng M, et al. The preablation monocyte/high density lipoprotein ratio predicts the late recurrence of paroxysmal atrial fibrillation after radiofrequency ablation. *BMC Cardiovasc Disord*. 2020;20(1).

50. Shim J, Park JH, Kim JY, et al. Impaired mobilization of bone marrow derived CD34 positive mononuclear cells is related to the recurrence of atrial fibrillation after radiofrequency catheter ablation. *Int J Cardiol*. 2013;162(3):179-183.

51. Cabrera-Bueno F, Medina-Palomo C, Ruiz-Salas A, et al. Serum levels of interleukin-2 predict the recurrence of atrial fibrillation after pulmonary vein ablation. *Cytokine*. 2015;73(1):74-78.

52. Ravassa S, Ballesteros G, López B, et al. Combination of Circulating Type I Collagen-Related Biomarkers Is Associated With Atrial Fibrillation. *J Am Coll Cardiol*. 2019;73(12):1398-1410.

53. Kornej J, Schmidl J, Ueberham L, et al. Galectin-3 in patients with atrial fibrillation undergoing radiofrequency catheter ablation. *PloS One*. 2015;10(4):e0123574.

54. Clementy N, Benhenda N, Piver E, et al. Serum Galectin-3 Levels Predict Recurrences after Ablation of Atrial Fibrillation. *Sci Rep*. 2016;6:34357.

55. Uçar FM, Gucuk İpek E, Acar B, et al. Gamma-glutamyl transferase predicts recurrences of atrial fibrillation after catheter ablation. *Acta Cardiol*. 2016;71(2):205-210.

56. Miake J, Kato M, Ogura K, et al. Pre-ablation levels of brain natriuretic peptide are independently associated with the recurrence of atrial fibrillation after radiofrequency catheter ablation in patients with nonvalvular atrial fibrillation. *Heart Vessels*. 2019;34(3):517-526.

57. Qu X, Chen L, Sun L, et al. Serum relaxin level predicts recurrence of atrial fibrillation after radiofrequency catheter ablation. *Heart Vessels*. 2019;34(9):1543-1551.

58. Macías-Ruiz R, Jiménez-Jáimez J, Álvarez-López M, et al. Effect of pulmonary vein catheter ablation on kidney function in patients with atrial fibrillation. A prospective cohort study. *Rev Esp Cardiol Engl Ed*. 2020;73(6):471-478.

59. Tokuda M, Yamane T, Matsuo S, et al. Relationship between renal function and the risk of recurrent atrial fibrillation following catheter ablation. *Heart Br Card Soc*. 2011;97(2):137-142.

60. Kawaji T, Shizuta S, Aizawa T, et al. Renal function and outcomes in atrial fibrillation patients after catheter ablation. Shimosawa T, ed. *PLOS ONE*. 2020;15(11):e0241449.

61. Okawa K, Miyoshi T, Sogo M, et al. Improvement in renal and endothelial function after catheter ablation in patients with persistent atrial fibrillation. *J Cardiol*. 2020;76(6):610-617.

62. Li A, Chen Y, Wang W, Su L, Ling Z. Association of clinical predictors with recurrence of atrial fibrillation after catheter ablation. *Ann Noninvasive Electrocardiol*. 2020;25(6).

63. Canpolat U, Aytemir K, Yorgun H, et al. Usefulness of serum uric acid level to predict atrial fibrillation recurrence after cryoballoon-based catheter ablation. *Eur Eur Pacing Arrhythm Card Electrophysiol J Work Groups Card Pacing Arrhythm Card Cell Electrophysiol Eur Soc Cardiol*. 2014;16(12):1731-1737.

64. Lellouche N, Sacher F, Wright M, et al. Usefulness of C-reactive protein in predicting early and late recurrences after atrial fibrillation ablation. *Europace*. 2009;11(5):662-664.

65. Deng H, Shantsila A, Guo P, et al. Sex-related risks of recurrence of atrial fibrillation after ablation: Insights from the Guangzhou Atrial Fibrillation Ablation Registry. *Arch Cardiovasc Dis*. 2019;112(3):171-179.

66. Celik AI, Kanadasi M, Demir M, et al. Predictors of the paroxysmal atrial fibrillation recurrence following cryoballoon-based pulmonary vein isolation: Assessment of left atrial volume, left atrial volume index, galectin-3 level and neutrophil-to-lymphocyte ratio. *Indian Pacing Electrophysiol J*. 2019;19(1):9-14.

67. Choi J-I, Baek YS, Roh SY, Piccini JP, Kim Y-H. Chromosome 4q25 variants and biomarkers of myocardial fibrosis in patients with atrial fibrillation. *J Cardiovasc Electrophysiol*. 2019;30(10):1904-1913.

68. Wu C-H, Hu Y-F, Chou C-Y, et al. Transforming growth factor-β1 level and outcome after catheter ablation for nonparoxysmal atrial fibrillation. *Heart Rhythm*. 2013;10(1):10-15.

69. Canpolat U, Aytemir K, Yorgun H, et al. Role of Preablation Neutrophil/Lymphocyte Ratio on Outcomes of Cryoballoon-Based Atrial Fibrillation Ablation. *Am J Cardiol*. 2013;112(4):513-519.

70. Gunes HM, Babur Guler G, Guler E, et al. Relationship between serum osteopontin level and atrial fibrillation recurrence in patients undergoing cryoballoon catheter ablation. *Turk Kardiyol Dernegi Arsivi Turk Kardiyol Derneginin Yayin Organidir*. 2017;45(1):26-32.

71. Yano M, Egami Y, Yanagawa K, et al. Predictors of recurrence after pulmonary vein isolation in patients with normal left atrial diameter. *J Arrhythmia*. 2020;36(1):75-81.

72. Aksu T, Golcuk SE, Guler TE, Yalin K, Erden I. Prediction of mid-term outcome after cryo-balloon ablation of atrial fibrillation using post-procedure high-sensitivity troponin level. *Cardiovasc J Afr*. 2015;26(4):165-170.
