# Supplementary material for: Biomarkers as predictors of recurrence of atrial fibrillation post ablation: an updated and expanded systematic review and meta-analysis
Source: Clin Res Cardiol. 2022 Jan 9;111(6):680–91. doi: 10.1007/s00392-021-01978-w (PMC9151522; doi:10.1007/s00392-021-01978-w)
Supplement: Supplementary file 2 — Supplementary file2 (DOCX 2626 KB) [file 392_2021_1978_MOESM2_ESM.docx]

**Supplementary Figures Legend**

1. Supplementary Figure 1: Forrest plot showing association between baseline biomarkers without outliers [BNP (S1A), NT-proBNP (S1B), hsCRP (S1C), eGFR (S1D)] and AF recurrence post-catheter ablation.
2. Supplementary Figure 2: Forrest plot showing association between baseline lipid profile markers [Cholesterol (S2A), LDL (S2B), HDL (S2C), Triglycerides (S1D)] and AF recurrence post-catheter ablation.
3. Supplementary Figure 3: Forrest plot showing association between baseline inflammatory markers [CRP (S3A), NLR (S3B), TNF (S3C), TGF (S3D)] and AF recurrence post-catheter ablation.
4. Supplementary Figure 4: Forrest plot showing association between baseline fibrosis markers [Galectin (S4A), TIMP (S4B)] and AF recurrence post-catheter ablation. SD: standard deviation.
5. Supplementary Figure 5: Forrest plot showing association between baseline biomarkers: creatinine (S4A), troponin (S5B), HbA1c (S5C) and AF recurrence post-catheter ablation.
6. Supplementary Figure 6: Subgroup analysis assessing impact of AF type [(a) paf, (b) paf+persaf, (c) paf+persaf+lspaf] on baseline BNP in recurrence and non-recurrence groups.
7. Supplementary Figure 7: Funnel plots for BNP (S7A), NT-proBNP (S7B), hsCRP (S7C), WBC (S7D) and eGFR (S7E).

Supplementary Figure 1: Forrest plot showing association between baseline biomarkers without outliers [BNP (S1A), NT-proBNP (S1B), hsCRP (S1C), eGFR (S1D)] and AF recurrence post-catheter ablation. TE: Estimate of effect size, SE: standard error of effect size, CI: confidence interval

Supplementary Figure 2: Forrest plot showing association between baseline lipid profile markers [Cholesterol (S2A), LDL (S2B), HDL (S2C), Triglycerides (S2D)] and AF recurrence post-catheter ablation. TE: Estimate of effect size, SE: standard error of effect size, CI: confidence interval.

Supplementary Figure 3: Forrest plot showing association between baseline inflammatory markers [CRP (S3A), NLR (S3B), TNF (S3C), TGF (S3D)] and AF recurrence post-catheter ablation. TE: Estimate of effect size, SE: standard error of effect size, CI: confidence interval.

Supplementary Figure 4: Forrest plot showing association between baseline fibrosis markers [Galectin (S4A), TIMP (S4B)] and AF recurrence post-catheter ablation. TE: Estimate of effect size, SE: standard error of effect size, CI: confidence interval.

Supplementary Figure 5: Forrest plot showing association between baseline biomarkers: creatinine (S5A), troponin (S5B), HbA1c (S5C) and AF recurrence post-catheter ablation. TE: Estimate of effect size, SE: standard error of effect size, CI: confidence interval.

Supplementary Figure 6: Subgroup analysis assessing impact of AF type [(a) paf, (b) paf+persaf, (c) paf+persaf+lspaf (d) paf+nonpaf (e) persaf (f) nonpaf] on baseline BNP in recurrence and non-recurrence groups. TE: Estimate of effect size, SE: standard error of effect size, CI: confidence interval

paf: paroxysmal AF, persAF: persistent AF; lspaf: long-standing persistent AF

Supplementary Figure 7: Funnel plots for BNP (S7A), NT-proBNP (S7B), hsCRP (S7C), WBC (S7D) and eGFR (S7E).
