# Supplementary material for: Biomarkers as predictors of recurrence of atrial fibrillation post ablation: an updated and expanded systematic review and meta-analysis
Source: Clin Res Cardiol. 2022 Jan 9;111(6):680–91. doi: 10.1007/s00392-021-01978-w (PMC9151522; doi:10.1007/s00392-021-01978-w)
Supplement: Supplementary file 3 — Supplementary file3 (DOCX 87 KB) [file 392_2021_1978_MOESM3_ESM.docx]

# Supplementary Material

**Legend**

Supplementary Table 1: PRIMSA Checklist

Supplementary Table 2: Search strategy

Supplementary Table 3: PICO(TS) model

Supplementary Table 4: Inclusion and exclusion criteria

Supplementary Table 5: Baseline characteristics of papers included

Supplementary Table 6: Ablation type, lines, proportions of AF categories of papers included

Supplementary Table 7: Meta-regression

Supplementary Table 8: Bias assessment using QUIPS for papers included

**Supplementary Table 1: PRIMSA Checklist**

| **Section/topic** | **#** | **Checklist item** | **Reported on page #** |
| --- | --- | --- | --- |
| **TITLE** | | |  |
| Title | 1 | Identify the report as a systematic review, meta-analysis, or both. | 2 |
| **ABSTRACT** | | |  |
| Structured summary | 2 | Provide a structured summary including, as applicable: background; objectives; data sources; study eligibility criteria, participants, and interventions; study appraisal and synthesis methods; results; limitations; conclusions and implications of key findings; systematic review registration number. | 1 |
| **INTRODUCTION** | | |  |
| Rationale | 3 | Describe the rationale for the review in the context of what is already known. | 2 |
| Objectives | 4 | Provide an explicit statement of questions being addressed with reference to participants, interventions, comparisons, outcomes, and study design (PICOS). | 2 |
| **METHODS** | | |  |
| Protocol and registration | 5 | Indicate if a review protocol exists, if and where it can be accessed (e.g., Web address), and, if available, provide registration information including registration number. | NA |
| Eligibility criteria | 6 | Specify study characteristics (e.g., PICOS, length of follow-up) and report characteristics (e.g., years considered, language, publication status) used as criteria for eligibility, giving rationale. | 2 |
| Information sources | 7 | Describe all information sources (e.g., databases with dates of coverage, contact with study authors to identify additional studies) in the search and date last searched. | 2,3 |
| Search | 8 | Present full electronic search strategy for at least one database, including any limits used, such that it could be repeated. | 2 |
| Study selection | 9 | State the process for selecting studies (i.e., screening, eligibility, included in systematic review, and, if applicable, included in the meta-analysis). | 2,3 |
| Data collection process | 10 | Describe method of data extraction from reports (e.g., piloted forms, independently, in duplicate) and any processes for obtaining and confirming data from investigators. | 3 |
| Data items | 11 | List and define all variables for which data were sought (e.g., PICOS, funding sources) and any assumptions and simplifications made. | 2,3 |
| Risk of bias in individual studies | 12 | Describe methods used for assessing risk of bias of individual studies (including specification of whether this was done at the study or outcome level), and how this information is to be used in any data synthesis. | 3 |
| Summary measures | 13 | State the principal summary measures (e.g., risk ratio, difference in means). | 3 |
| Synthesis of results | 14 | Describe the methods of handling data and combining results of studies, if done, including measures of consistency (e.g., I^2^) for each meta-analysis. | 3 |
| Risk of bias across studies | 15 | Specify any assessment of risk of bias that may affect the cumulative evidence (e.g., publication bias, selective reporting within studies). | 3 |
| Additional analyses | 16 | Describe methods of additional analyses (e.g., sensitivity or subgroup analyses, meta-regression), if done, indicating which were pre-specified. | 3,4 |
| **RESULTS** | | |  |
| Study selection | 17 | Give numbers of studies screened, assessed for eligibility, and included in the review, with reasons for exclusions at each stage, ideally with a flow diagram. | 4 |
| Study characteristics | 18 | For each study, present characteristics for which data were extracted (e.g., study size, PICOS, follow-up period) and provide the citations. | 5, 6, Supp. table 1 |
| Risk of bias within studies | 19 | Present data on risk of bias of each study and, if available, any outcome level assessment (see item 12). | Supp table 3 |
| Results of individual studies | 20 | For all outcomes considered (benefits or harms), present, for each study: (a) simple summary data for each intervention group (b) effect estimates and confidence intervals, ideally with a forest plot. | 6-11 |
| Synthesis of results | 21 | Present results of each meta-analysis done, including confidence intervals and measures of consistency. | 6-11 |
| Risk of bias across studies | 22 | Present results of any assessment of risk of bias across studies (see Item 15). | 6-11 |
| Additional analysis | 23 | Give results of additional analyses, if done (e.g., sensitivity or subgroup analyses, meta-regression [see Item 16]). | 11 |
| **DISCUSSION** | | |  |
| Summary of evidence | 24 | Summarize the main findings including the strength of evidence for each main outcome; consider their relevance to key groups (e.g., healthcare providers, users, and policy makers). | 11-14 |
| Limitations | 25 | Discuss limitations at study and outcome level (e.g., risk of bias), and at review-level (e.g., incomplete retrieval of identified research, reporting bias). | 14 |
| Conclusions | 26 | Provide a general interpretation of the results in the context of other evidence, and implications for future research. | 11-14 |
| **FUNDING** | | |  |
| Funding | 27 | Describe sources of funding for the systematic review and other support (e.g., supply of data); role of funders for the systematic review. | 14 |

**Supplementary Table 2: Search strategy**

| Initial search strategy conducted in Pubmed/Medline and Embase | | |
| --- | --- | --- |
| # | Search term | Results |
| 1 | atrial fibrillation.af. | 249696 |
| 2 | ablation.af. | 288851 |
| 3 | biomarker*.af. | 1139256 |
| 4 | 1 and 2 and 3 | 616 |
| Articles from the initial search strategy were reviewed to identify biomarkers that have been studied in atrial fibrillation. These biomarkers were collated and search repeated in Pubmed/Medline, Embase, Web of Science and Scopus | | |
| # | Search term | Results |
| 1 | atrial fibrillation | 249696 |
| 2 | ablation | 288851 |
| 3 | biomarker* | 1139256 |
| 4 | proadrenomedullin OR osteoprotegerin or interleukin or CTGF or glycogen phosphorylase BB or osteoprotegerin or ST2 or RANKL or proadrenomedullin or NGF or YKL-40 or monocyte or IFN or heat shock protein or fibrocyte or Fas or E-selectin or CGA or ferritin or NOx or TIMP or osteopontin or apelin or aldosterone or RAAS or telomere length or TRAP or hFABP or thyroid or genes or FGF or VCAM or VEGF or MCP or peroxynitrite or relaxin or myeloperoxidase or cholesterol or adiponectin or myoglobin or red cell or HbA1c or fibrinogen or endothelin or homocysteine or uric acid or NLR or plasminogen or norepinephrine or lipoprotein or P-selectin or NTproANP or NT-proANP or MR-proANP or vWF or oxidative stress or RNA or mRNA or microRNA or miR or d-dimer or TNF or TGF or galectin or ANP or platelet or NTproBNP or NT-proBNP or CKMB or collagen or troponin* or BNP or CRP or hsCRP or natriuretic peptide or cystatin or eGFR or chemokine or MMP or Neutrophil or Lymphocyte or GDF or urokinase or PIIINP or creatinine or CITP or ICTP or t-PA or plasminogen activator inhibitor or fibrinopeptide or thrombin antithrombin or soluble thrombomodulin or antiplasmin or urokinase-type plasmin-antiplasmin or white blood cell or thromboglobulin or HDL or LDL or triglyceride or TIMP or CD40 or ADMA or IL6 or IL1 or IL2 or IL8 or IL10 or PAI or PAP or IL16 or IL4 or IL12 or Gal-3 or GDF15 or GDF-15 or Gal3 | 12205225 |
| 5 | 3 or 4 | 12764695 |
| 6 | 1 and 2 and 5 | 3838 |

**Supplementary Table 3: PICO(TS) model**

| Population | Adults aged 18 years and over with atrial fibrillation undergoing catheter ablation |
| --- | --- |
| Index prognostic factor(s) | Biomarker |
| Comparators | Not applicable |
| Outcome | Recurrence is defined as $\geq$30 seconds of arrhythmia following ablation, post blanking period of three months as per International guidelines (American/European) |
| Timing | Pre-procedural blood test |
| Setting | Hospital |

**Supplementary Table 4: Inclusion and exclusion criteria**

| Inclusion criteria | Exclusion criteria |
| --- | --- |
| (1) human studies  (2) atrial fibrillation ablation  (3) post-ablation recurrence assessed for $\boldsymbol{\geq}$3 months  (4) randomised controlled trials (RCT)  (5) prospective observational studies  (6) retrospective observational studies  (7) any biomarker assessed in a peripheral blood sample  (8) first ablation (RF/CB/standalone SA)  (9) recurrence of $\boldsymbol{\geq}$30secs atrial arrhythmia (AF, atrial flutter, atrial tachycardia (AT) assessed using 12-lead ECG or Holter ECG  (10) blanking period of 3 months  (11) mean (standard deviation) or median [range or interquartile range (IQR)] of biomarker within recurrence (R) and non-recurrence (NR) groups were provided | (1) abstracts  (2) case reports  (3) conference papers  (4) expert opinions, editorials, letter to editors  (5) non-English articles  (6) reviews  (7) concomitant surgical procedures (valvular replacement, coronary artery bypass graft) |

**Supplementary Table 5: Baseline characteristics of papers included**

| **Author** | **Year** | **Country** | **Follow-up (mean months)** | **Type of study** | **Total no. AF cohort** | **Control (non-AF)** | **Age (mean)** |  | **Gender (M%)** | **Holter** |
| --- | --- | --- | --- | --- | --- | --- | --- | --- | --- | --- |
|  |  |  |  |  |  |  |  | **Age (SD)** |  |  |
| Date T | 2006 | Japan | 14 | Pro | 53 | 21 | 53 | 10 | 89 | Y |
| Yamada T | 2006 | Japan | >3 | Pro | 66 | NA | 61 | 10 | 82 | Y |
| Letsas K | 2009 | Germany | 13 | Ret | 72 | NA | 55 | 9 | 81 | Y |
| Nakazawa Y | 2009 | Japan | 6 | Pro | 51 | NA | 58 | 11 | 88 | Y |
| Lellouche N | 2009 | France | 14 | Ret | 125 | NA | 54 | 11 | 78 | N |
| Nilsson B | 2009 | Denmark | 12 | RCT | 51 | NA | 56 | 11 | 63 | Y |
| Henningsen K | 2009 | Denmark | 12 | RCT | 46 | NA | 55 | 11 | 63 | Y |
| Liu J | 2010 | China | 23 | Ret | 121 | NA | 55 | 12 | 77 | Y |
| Tokuda M | 2010 | Japan | 37 | Ret | 224 | NA | 55 | 12 | 83 | Y |
| den Uijl D | 2011 | Netherlands | 7 | Pro | 87 | NA | 55 | 9 | 80 | Y |
| Shin S | 2011 | Korea | 6 | Pro | 80 | 80 | 54 | 9 | 58 | y |
| Machino-Ohtsua T | 2011 | Japan | 34 | Pro | 155 | NA | 61 | 9 | 79 | Y |
| Naruse Y | 2011 | Japan | 32 | Pro | 221 | NA | 59 | 11 | 81 | Y |
| Okumura Y | 2011 | Japan | 14 | Pro | 50 | NA | 61 | 9 | 76 | Y |
| Wang H | 2012 | China | 22 | Pro | 158 | NA | 55 | 12 | 68 | Y |
| Kornej J | 2013 | Germany | 6 | Pro | 68 | NA | 59 | 11 | 65 | Y |
| Canpolat U | 2013 | Turkey | 29 | Pro | 251 | NA | 54 | 11 | 52 | N |
| Im S | 2013 | South Korea | 25 | NA | 499 | NA | 57 | 11 | 74 | N |
| Li S | 2013 | China | 12 | Ret | 288 | NA | 57 | 11 | 70 | Y |
| He X | 2013 | China | 9 | Ret | 330 | NA | 60 | 11 | 68 | Y |
| Zou C | 2013 | China | 12 | Ret | 76 | 77 | 65 | 9 | 51 | Y |
| Shim J | 2013 | Republic of Korea | 30 | Pro | 46 | 10 | 56 | 11 | 74 | Y |
| Wu C | 2013 | Taiwan | 11 | Pro | 46 | NA | 53 | 9 | 91 | Y |
| Canpolat U | 2014 | Turkey | 19 | Pro | 363 | NA | 54 | 11 | 53 | Y |
| Pillarisetti J | 2014 | USA | 12 | Pro | 88 | NA | 60 | 8 | 68 | Y |
| Guo X | 2014 | China | 31 | NA | 379 | NA | 50 | 7 | 73 | Y |
| Kimura T | 2014 | Japan | 10 | Pro | 44 | NA | 59 | 8 | - | Y |
| Qiong H | 2014 | China | 12 | Ret | 120 | NA | 52 | 12 | 59 | N |
| Aksu To | 2015 | Turkey | 7 | Pro | 57 | NA | 55 | 12 | 50 | y |
| Aksu T | 2015 | Turkey | 10 | Pro | 49 | NA | 58 | 12 | 51 | Y |
| Cabrera-Bueno F | 2015 | Spain | 19 | Pro | 44 | 10 | 58 | 10 | 73 | Y |
| Canpolat U | 2015 | Turkey | 20.6 | Pro | 402 | NA | 54 | 11 | 56 | Y |
| Parwani A | 2015 | Germany | 12 | Pro | 87 | NA | 62 | 9 | 66 | Y |
| Kornej J | 2015 | Germany | 6 | Pro | 105 | 14 | 62 | 9 | 65 | Y |
| Shaikh A | 2015 | USA | 6 | Ret | 161 | NA | 59 | 10 | 70 | Y |
| Wu X | 2015 | China | 17 | Pro | 50 | 46 | 50 | 8 | 96 | y |
| Uçar F | 2016 | Turkey | 20 | Ret | 102 | NA | 50 | 12 | 63 | Y |
| Clementy N | 2016 | France | 12 | Pro | 160 | NA | 61 | 10 | 71 | Y |
| Yanagisawa S | 2016 | Japan | 6 | Ret | 54 | NA | 60 | 13 | 85 | Y |
| Ma X | 2017 | China | 12 | Pro | 120 | NA | 64 | 7 | 60 | Y |
| Güneş H | 2017 | Turkey | 12 | Pro | 60 | NA | 54 | 11 | 60 | Y |
| Shiozawa T | 2017 | Japan | 19.2 | Pro | 78 | NA | 59 | 9 | 81 | Y |
| Kishima H | 2017 | Japan | 12 | Pro | 113 | 38 | 65 | 10 | 65 | Y |
| Deng H | 2018 | China | 20.7 | Ret | 1410 | NA | 57 | 12 | 68 | Y |
| Luetkens J | 2018 | Germany | 12 | Pro | 61 | NA | 60 | 13 | 66 | Y |
| Clementy N | 2018 | France | 12 | Pro | 75 | NA | 63 | 10 | 81 | Y |
| Liu L | 2019 | China | 12 | Pro | 384 | NA | 66 | 9 | 59 | Y |
| Qu X | 2019 | China | 18 | Pro | 248 | NA | 66 | 11 | 63 | Y |
| Bazoukis G | 2019 | Greece | 26 | Ret | 346 | NA | 59 | NA | 65 | Y |
| Deng H | 2019 | China | 12 | Ret | 450 | NA | 58 | 12 | 68 | Y |
| Tamura S | 2019 | Japan | 15 | Ret | 227 | NA | 66 | 10 | 69 | Y |
| Miake J | 2019 | Japan | 24 | Pro | 254 | NA | 66 | 9 | 72 | Y |
| Su C | 2019 | China | 14 | Pro | 282 | NA | 65 | 11 | 54 | Y |
| Celik A | 2019 | Turkey | 12 | Pro | 50 | NA | 50 | 13 | 56 | Y |
| Ravassa S | 2019 | Spain | 12 | Pro | 150 | NA | 64 | 10 | 67 | Y |
| Yano M | 2019 | Japan | >3 | Pro | 168 | NA | 64 | 10 | 64 | Y |
| Namino F | 2019 | Japan | 6 | Ret | 101 | NA | 62 | 9 | 80 | Y |
| Choi J | 2019 | Republic of Korea | 37 | Ret | 137 | 70 | 56 | 12 | 83 | Y |
| Xu M | 2020 | China | 6 | Ret | 233 | NA | 63 | 10 | 84 | Y |
| Okawa K (PAF) | 2020 | Japan | 12 | Pro | 229 | NA | 68 | 8 | 62 | Y |
| Liu H | 2020 | China | 13 | Pro | 266 | NA | 61 | 9 | 55 | Y |
| Okawa K (PersAF) | 2020 | Japan | 12 | Pro | 129 | NA | 68 | 8 | 73 | Y |
| Yano M | 2020 | Japan | 12 | Pro | 369 | NA | 70 | 8 | 40 | Y |
| Du W | 2020 | China | 12 | Ret | 108 | NA | 63 | 8 | 54 | Y |
| Huang Z | 2020 | China | 12 | Ret | 422 | NA | 64 | 10 | 65 | Y |
| Wei Y | 2020 | China | 24 | Pro | 150 | NA | 64 | 11 | 57 | Y |
| Li A | 2020 | China | 12 | Pro | 257 | NA | 61 | 12 | 53 | Y |
| Macias-Ruiz R | 2020 | Spain | 12 | Pro | 124 | NA | 55 | 10 | 69 | Y |
| Kawaji T | 2020 | Japan | 61 | Pro | 791 | NA | 65 | 10 | 69 | Y |
| Chen S | 2020 | China | 25 | Ret | 125 | NA | 61 | 9 | 55 | Y |
| Oka T | 2020 | Japan | 36 | Ret | 292 | NA | 62 | 10 | 72 | Y |
| Can V | 2021 | Turkey | 12 | Pro | 101 | 60 | 60 | NA | 36 | Y |
| Nakamura K | 2021 | Japan | 12 | Pro | 194 | NA | 65 | 11 | 77 | Y |
| Yang Z | 2021 | China | >3 | Ret | 215 | NA | 63 | 10 | 84 | Y |

*M: Male; N: No; NA: not available; Pro: prospective; RCT: randomised controlled trial; Ret: retrospective; Y: yes*

**Supplementary Table 6: Ablation type, lines, proportions of AF categories of papers included**

| **Author** | **Year** | **Type of ablation** | **Ablation Lines** | | | | | **Total no. AF cohort** | **Type of AF** | | | | **Recurrence (%)** |
| --- | --- | --- | --- | --- | --- | --- | --- | --- | --- | --- | --- | --- | --- |
|  |  |  | **PVI** | **Linear lesions** | **Mitral isthmus** | **CFAE** | **CTI** |  | **PAF %** | **PersAF %** | **LSPAF %** | **Non-PAF %** |  |
| Date T | 2006 | RF | 1 | 0 | 0 | 0 | 0 | 53 | 100 | - | - | - | 40 |
| Yamada T | 2006 | RF | 1 | 0 | 0 | 0 | 0 | 66 | 100 | - | - | - | 47 |
| Letsas K | 2009 | RF | 0 | 1 | 0 | 0 | 0 | 72 | 64 | 36 | - | - | 39 |
| Nakazawa Y | 2009 | RF | 1 | 0 | 0 | 0 | 1 | 51 | 76 | 24 | - | - | 47 |
| Lellouche N | 2009 | RF | 1 | 1 | 1 | 0 | 1 | 125 | 52 | - | - | 48 | 47 |
| Nilsson B | 2009 | RF | 1 | 0 | 0 | 0 | 0 | 51 | 67 | 33 | - | - | 75 |
| Henningsen K | 2009 | RF | 1 | 0 | 0 | 0 | 0 | 46 | 67 | 33 | - | - | 83 |
| Liu J | 2010 | RF | 1 | 1 | 1 | 1 | 1 | 121 | 64 | 36 | - | - | 30 |
| Tokuda M | 2010 | RF | 1 | 0 | 0 | 0 | 0 | 224 | 100 | - | - | - | 64 |
| den Uijl D | 2011 | RF | 1 | 0 | 0 | 0 | 0 | 87 | 89 | 10 | - | - | 24 |
| Shin S | 2011 | RF | 1 | 0 | 0 | 1 | 1 | 80 | 76 | 24 | - | - | 28 |
| Machino-Ohtsua T | 2011 | RF | 1 | 1 | 0 | 0 | 1 | 155 | 65 | 35 | - | - | 29 |
| Naruse Y | 2011 | RF | 1 | 1 | 0 | 1 | 0 | 221 | 57 | 43 | - | - | 39 |
| Okumura Y | 2011 | RF | 1 | 0 | 0 | 1 | 0 | 50 | 56 | 36 | 8 | - | 42 |
| Wang H | 2012 | RF | 1 | 1 | 1 | 0 | 0 | 158 | 65 | 35 | - | - | 45 |
| Kornej J | 2013 | RF | 0 | 1 | 1 | 0 | 0 | 68 | 59 | 41 | - | - | 18 |
| Canpolat U | 2013 | CB | 1 | 0 | 0 | 0 | 0 | 251 | 80 | 20 | - | - | 24 |
| Im S | 2013 | RF | NA | NA | NA | NA | NA | 499 | 53 | 47 | - | - | 24 |
| Li S | 2013 | RF | 1 | 0 | 0 | 0 | 1 | 288 | 100 | - | - | - | 28 |
| He X | 2013 | RF | 1 | 0 | 0 | 0 | 0 | 330 | 100 | - | - | - | 32 |
| Zou C | 2013 | RF | 1 | 1 | 1 | 0 | 0 | 76 | 57 | 43 | - | - | 33 |
| Shim J | 2013 | RF | 1 | 1 | 1 | 0 | 1 | 46 | 57 | 43 | - | - | 52 |
| Wu C | 2013 | RF | 1 | 0 | 0 | 1 | 0 | 46 | - | - | - | 100 | 65 |
| Canpolat U | 2014 | CB | 1 | 0 | 0 | 0 | 0 | 363 | 100 | - | - | - | 19 |
| Pillarisetti J | 2014 | RF | 1 | 1 | 0 | 1 | 1 | 88 | 33 | 64 | 3 | - | 22 |
| Guo X | 2014 | RF | 1 | 1 | 1 | 0 | 1 | 379 | 60 | - | - | 40 | 33 |
| Kimura T | 2014 | RF | 1 | 0 | 0 | 1 | 0 | 44 | 70 | 30 | - | - | 34 |
| Qiong H | 2014 | RF | 1 | 0 | 0 | 1 | 0 | 120 | - | - | - | 100 | 44 |
| Aksu To | 2015 | RF, CB | 1 | 0 | 0 | 1 | 0 | 57 | 100 | 0 | 0 | 0 | 12 |
| Aksu T | 2015 | CB | 1 | 0 | 0 | 0 | 0 | 49 | 100 | - | - | - | 14 |
| Cabrera-Bueno F | 2015 | RF | 1 | 0 | 0 | 0 | 1 | 44 | 100 | - | - | - | 16 |
| Canpolat U | 2015 | CB | 1 | 0 | 0 | 0 | 0 | 402 | 81 | 19 | - | - | 24 |
| Parwani A | 2015 | RF | 1 | 1 | 1 | 0 | 0 | 87 | 54 | 46 | - | - | 29 |
| Kornej J | 2015 | RF | 1 | 0 | 0 | 0 | 0 | 105 | 52 | - | - | 48 | 34 |
| Shaikh A | 2015 | RF, CB | 1 | 0 | 0 | 0 | 0 | 161 | 60 | 40 | - | - | 48 |
| Wu X | 2015 | RF | 1 | 1 | 1 | 0 | 1 | 50 | 0 | 100 | 0 | 0 | 64 |
| Uçar F | 2016 | CB | 1 | 0 | 0 | 0 | 0 | 102 | 100 | - | - | - | 19 |
| Clementy N | 2016 | RF | 1 | 1 | 1 | 1 | 0 | 160 | 55 | - | - | 45 | 34 |
| Yanagisawa S | 2016 | RF | 1 | 1 | 0 | 1 | 0 | 54 | 30 | 48 | 22 | - | 35 |
| Ma X | 2017 | RF | 1 | 0 | 0 | 0 | 0 | 120 | 46 | 54 | - | - | 33 |
| Güneş H | 2017 | CB | 1 | 0 | 0 | 0 | 0 | 60 | 78 | 22 | - | - | 37 |
| Shiozawa T | 2017 | RF | 1 | 1 | 0 | 0 | 0 | 78 | 64 | - | - | 36 | 37 |
| Kishima H | 2017 | RF | 0 | 1 | 0 | 0 | 1 | 113 | 48 | 52 | - | - | 38 |
| Deng H | 2018 | RF, CB | 1 | 1 | 1 | 1 | 1 | 1410 | 77 | - | - | 23 | 26 |
| Luetkens J | 2018 | CB | 1 | 0 | 0 | 0 | 0 | 61 | 66 | 34 | - | - | 33 |
| Clementy N | 2018 | RF | 1 | 1 | 1 | 1 | 0 | 75 | 100 | 100 | - | - | 33 |
| Liu L | 2019 | CB | 1 | 0 | 0 | 0 | 0 | 384 | 86 | 14 | 0 | 0 | 21 |
| Qu X | 2019 | RF | 1 | 1 | 1 | 0 | 0 | 248 | 51 | 49 | - | - | 21 |
| Bazoukis G | 2019 | CB | 1 | 0 | 0 | 0 | 0 | 346 | 62 | 35 | 3 | 0 | 23 |
| Deng H | 2019 | CB | 1 | 1 | 1 | 1 | 1 | 450 | 77 | 0 | 0 | 23 | 25 |
| Tamura S | 2019 | RF, CB | 1 | 0 | 0 | 0 | 0 | 227 | 56 | 44 | 0 | 0 | 25 |
| Miake J | 2019 | RF | 1 | 0 | 0 | 0 | 1 | 254 | 69 | 31 | - | - | 26 |
| Su C | 2019 | RF | 1 | 1 | 1 | 0 | 1 | 282 | 65 | - | - | 35 | 28 |
| Celik A | 2019 | RF | 1 | 0 | 0 | 0 | 0 | 50 | 100 | - | - | - | 28 |
| Ravassa S | 2019 | RF | 1 | 0 | 0 | 0 | 0 | 150 | 57 | 43 | - | - | 29 |
| Yano M | 2019 | RF | 1 | 0 | 0 | 0 | 1 | 168 | 93 | 0 | 0 | 7 | 30 |
| Namino F | 2019 | RF | 0 | 0 | 0 | 1 | 0 | 101 | 53 | 36 | 11 | 0 | 46 |
| Choi J | 2019 | RF | 1 | 1 | 0 | 0 | 1 | 137 | 0 | 0 | 0 | 100 | 56 |
| Xu M | 2020 | RF | 1 | 0 | 0 | 0 | 0 | 233 | 100 | 0 | 0 | 0 | 18 |
| Okawa K (PAF) | 2020 | RF | 1 | 0 | 0 | 0 | 0 | 229 | 100 | 0 | 0 | 0 | 18 |
| Liu H | 2020 | RF | 1 | NA | NA | NA | NA | 266 | 64 | 0 | 0 | 36 | 20 |
| Okawa K (PersAF) | 2020 | RF | 1 | 0 | 0 | 0 | 0 | 129 | 0 | 100 | 0 | 0 | 20 |
| Yano M | 2020 | RF | 1 | 0 | 0 | 0 | 0 | 369 | 100 | 0 | 0 | 0 | 22 |
| Du W | 2020 | RF | 1 | NA | NA | 1 | NA | 108 | 66 | 34 | 0 | 0 | 22 |
| Huang Z | 2020 | NA | NA | NA | NA | NA | NA | 422 | 67 | 33 | 0 | 0 | 23 |
| Wei Y | 2020 | RF | 1 | 1 | NA | NA | NA | 150 | 59 | 41 | 0 | 0 | 25 |
| Li A | 2020 | RF | NA | NA | NA | NA | NA | 257 | 68 | 0 | 0 | 32 | 31 |
| Macias-Ruiz R | 2020 | RF, CB | 1 | 0 | 0 | 0 | 0 | 124 | 60 | 40 | 0 | 0 | 32 |
| Kawaji T | 2020 | RF | 1 | 1 | 0 | 1 | 1 | 791 | 69 | 0 | 0 | 31 | 34 |
| Chen S | 2020 | RF | 1 | 0 | 0 | 0 | 0 | 125 | 100 | 0 | 0 | 0 | 38 |
| Oka T | 2020 | RF | 1 | 1 | 1 | 1 | 1 | 292 | 100 | 0 | 0 | 0 | 46 |
| Can V | 2021 | CB | 1 | 0 | 0 | 0 | 0 | 101 | 100 | 0 | 0 | 0 | 20 |
| Nakamura K | 2021 | RF | 1 | 0 | 0 | 0 | 0 | 194 | 66 | 0 | 0 | 34 | 20 |
| Yang Z | 2021 | RF | 1 | 1 | 1 | 1 | 1 | 215 | 0 | 100 | 0 | 0 | 26 |

*CB: cryoballoon; CFAE: complex fractionated atrial electrogram; CTI: cavotricuspid isthmus; LSPAF: long standing persistent AF; NA: not available; PAF: paroxysmal AF; PersAF: persistent AF; PVI: pulmonary vein isolation; RF: radiofrequency.*

**Supplementary Table 7: Meta-regression**

| *Meta-regression (R^2^)* | *Age* | *Sex* | *Type of AF* | *Year of publication* | Ablation strategy (PVI or PVI+) | Follow-up (>3 months) |
| --- | --- | --- | --- | --- | --- | --- |
| *BNP* | *20.42* | *0* | *55.74* | *0* | 0 | 0 |
| *NT-proBNP* | *0* | *0* | *0* | *21.13* | 0 | 0 |
| *eGFR* | *0* | *8.97* | *5.37* | *0* | 0 | 0 |
| *hsCRP* | *0* | *0* | *0* | *0* | 0 | 0 |
| *WBC* | *2.19* | *0* | *0* | *0* | 0 | 0 |

*R2 statistic reported (proportion of variance that is explained by co-variate). Significance tests for residual heterogeneity were p<0.01.*

**Supplementary Table 8: Bias assessment using QUIPS for papers included**

| **Author** | **Year** | **QUIPS Bias Assessment** | | | | | | |
| --- | --- | --- | --- | --- | --- | --- | --- | --- |
|  |  | **Study Participation** | **Study Attrition** | **Prognostic Factor Measurement** | **Outcome Measurement** | **Study Confounding** | **Statistical Analysis & Reporting** | **Overall Risk of Bias** |
| **Date T** | **2006** | Moderate | Low | Low | Low | Moderate | Moderate | **Moderate** |
| **Yamada T** | **2006** | Moderate | Low | Low | Moderate | Moderate | Low | **Moderate** |
| **Henningsen K** | **2009** | Low | Low | Low | Low | Moderate | Low | Moderate |
| **Lellouche N** | **2009** | Moderate | Moderate | Low | High | High | Moderate | High |
| **Letsas K** | **2009** | Low | Low | Low | Low | Low | Low | **Low** |
| **Nakazawa Y** | **2009** | Low | Low | Low | Low | Low | Low | **Low** |
| **Nilsson B** | **2009** | Low | Low | Low | Moderate | Moderate | Low | Moderate |
| **Liu J** | **2010** | Low | Low | Low | Low | Moderate | Low | Moderate |
| **Tokuda M** | **2010** | Low | Low | Moderate | Moderate | Low | Low | Moderate |
| **den Uijl D** | **2011** | Moderate | Moderate | Low | Low | Low | Low | Moderate |
| **Machino-Ohtsua T** | **2011** | Low | Low | Low | Low | Low | Low | **Low** |
| **Naruse Y** | **2011** | Moderate | Low | Moderate | Low | Low | Low | **Moderate** |
| **Okumura Y** | **2011** | Moderate | Low | Low | Low | Moderate | Low | **Moderate** |
| **Shin S** | **2011** | High | Low | Low | Low | Low | Low | High |
| **Wang H** | **2012** | Low | Low | Low | Low | Low | Low | **Low** |
| **Canpolat U** | **2013** | Low | Low | Low | Low | Low | Low | **Low** |
| **He X** | **2013** | Low | Moderate | Low | Low | Low | Low | Moderate |
| **Im S** | **2013** | Moderate | Low | Low | High | Low | Low | High |
| **Kornej J** | **2013** | Low | Low | Low | Low | Low | Low | **Low** |
| **Li S** | **2013** | Low | Low | Low | Low | Low | Low | **Low** |
| **Shim J** | **2013** | High | Low | Low | Low | Low | Low | High |
| **Wu C** | **2013** | Moderate | Low | Low | Low | Low | Moderate | Moderate |
| **Zou C** | **2013** | Moderate | Low | Low | Low | Low | Low | Moderate |
| **Canpolat U** | **2014** | Low | Low | Low | Low | Low | Low | **Low** |
| **Guo X** | **2014** | Low | Low | Low | Low | Low | Low | **Low** |
| **Kimura T** | **2014** | Moderate | Low | Low | Low | Low | Low | Moderate |
| **Pillarisetti J** | **2014** | Low | Low | Low | Low | Moderate | Low | Moderate |
| **Qiong H** | **2014** | Moderate | Low | Low | Moderate | High | Moderate | High |
| **Aksu T** | **2015** | Low | Low | Low | Low | Low | Low | **Low** |
| **Aksu To** | **2015** | Low | Low | Low | Low | Low | Low | **Low** |
| **Cabrera-Bueno F** | **2015** | Low | Low | Low | Low | Low | Low | **Low** |
| **Canpolat U** | **2015** | Low | Low | Low | Low | Low | Low | **Low** |
| **Kornej J** | **2015** | High | Moderate | Low | Low | Low | Moderate | High |
| **Parwani A** | **2015** | Moderate | Low | Low | Low | Low | Low | Moderate |
| **Shaikh A** | **2015** | Low | Low | Low | Low | Moderate | Low | Moderate |
| **Wu X** | **2015** | Low | Low | Low | Low | Low | Low | **Low** |
| **Clementy N** | **2016** | Low | Low | Low | Low | Low | Low | **Low** |
| **Uçar F** | **2016** | Moderate | Low | Low | Low | Moderate | Low | Moderate |
| **Yanagisawa S** | **2016** | Moderate | Low | Low | Low | Low | Low | Moderate |
| **Güneş H** | **2017** | High | Low | Low | Low | Low | Low | High |
| **Kishima H** | **2017** | Moderate | Low | Low | Low | Low | Low | Moderate |
| **Ma X** | **2017** | Moderate | Low | Low | Low | Low | Low | Moderate |
| **Shiozawa T** | **2017** | Moderate | Low | Low | Low | Low | Low | Moderate |
| **Clementy N** | **2018** | Low | Low | Low | Low | Low | Low | **Low** |
| **Deng H** | **2018** | Low | Low | Low | Moderate | Moderate | Low | Moderate |
| **Luetkens J** | **2018** | Moderate | Low | Low | Low | Moderate | Low | Moderate |
| **Miake J** | **2018** | Low | Low | Low | Low | Low | Low | **Low** |
| **Bazoukis G** | **2019** | Low | Low | Moderate | Moderate | High | Moderate | High |
| **Celik A** | **2019** | High | Low | Low | Low | Moderate | Moderate | High |
| **Choi J** | **2019** | Low | Low | Low | Low | Moderate | Low | Moderate |
| **Deng H** | **2019** | Low | Low | Moderate | Low | Moderate | Low | Moderate |
| **Liu L** | **2019** | Low | Moderate | Low | Low | Low | Moderate | **Moderate** |
| **Namino F** | **2019** | Moderate | Low | Low | High | High | High | High |
| **Qu X** | **2019** | Low | Low | Low | Low | Low | Low | **Low** |
| **Ravassa S** | **2019** | Low | Low | Moderate | Low | Low | Low | Moderate |
| **Su C** | **2019** | Low | Low | Low | Low | Low | Low | **Low** |
| **Tamura S** | **2019** | Low | Low | Low | Low | Low | Low | **Low** |
| **Yano M** | **2019** | Low | Low | Moderate | Low | Low | Low | Moderate |
| **Macias-Ruiz R** | **2020** | Low | Low | Low | Low | Moderate | Low | Moderate |
| **Xu M** | **2020** | High | Low | Low | Low | Low | Low | High |
| **Chen S** | **2020** | High | Low | Moderate | Moderate | Moderate | Low | High |
| **Du W** | **2020** | Low | Low | Low | Moderate | Moderate | Low | Moderate |
| **Huang Z** | **2020** | Low | Low | Low | Moderate | Low | Low | Moderate |
| **Kawaji T** | **2020** | Low | Moderate | Moderate | High | Moderate | Low | High |
| **Li A** | **2020** | High | Low | Low | Low | Moderate | Low | High |
| **Liu H** | **2020** | Low | Moderate | Low | Low | Moderate | Low | Moderate |
| **Oka T** | **2020** | Low | Low | Low | Low | Moderate | Low | Moderate |
| **Okawa K** | **2020** | Low | Moderate | Low | Moderate | Low | Low | Moderate |
| **Wei Y** | **2020** | Low | Low | Low | Low | Moderate | Low | Moderate |
| **Yano M** | **2020** | Low | Low | Low | Low | Moderate | Low | Moderate |
| **Can V** | **2021** | Low | Low | Low | Moderate | Moderate | Low | Moderate |
| **Nakamura K** | **2021** | Low | Low | Low | Low | Moderate | Low | Moderate |
| **Yang Z** | **2021** | High | Low | Low | Moderate | Moderate | Low | High |
